# Two contrasting atmospheric circulation patterns linked to Kuroshio Extension variability

**DOI:** 10.1126/sciadv.aee9489

**Published:** 2026-08-07

**Authors:** Se-Yong Song, Samantha Stevenson, Emanuele Di Lorenzo, Antonietta Capotondi, Niklas Schneider, Youngji Joh

**Affiliations:** ^1^ Earth Research Institute, University of California, Santa Barbara, Santa Barbara, CA, USA.; ^2^ Bren School of Environmental Sciences and Management, University of California at Santa Barbara, Santa Barbara, CA, USA.; ^3^ Department of Earth, Environmental, and Planetary Sciences, Brown University, Providence, RI, USA.; ^4^ National Oceanic and Atmospheric Administration Physical Sciences Laboratory, Boulder, CO, USA.; ^5^ Cooperative Institute for Research in Environmental Sciences, University of Colorado Boulder, Boulder, CO, USA.; ^6^ Department of Oceanography and International Pacific Research Center and Advanced Institute for Marine Ecosystem Change, University of Hawai‘i at Mānoa, Honolulu, HI, USA.; ^7^ Geophysical Fluid Dynamics Laboratory/NOAA, Princeton, NJ, USA.

## Abstract

The influence of Kuroshio Extension (KE) variability on North Pacific atmospheric circulation has been increasingly recognized in high-resolution observations and models. While the role of fine-scale air-sea interactions has been highlighted, the dynamics underlying KE atmospheric impacts remain elusive. Here, based on observational analyses and a suite of model simulations, we report two contrasting atmospheric circulation patterns that arise from temporal variations in KE boundary forcing on the atmosphere. During the late 20th century (1979–1999), the positive KE phase, along with a northward shifted Kuroshio SST front, anchors the Pacific storm track farther poleward accompanied by an anomalous anticyclonic circulation. In contrast, during the early 21st century (2000–2022), KE variability exhibits stronger linkages with central tropical Pacific SST that drives a southward shift of the storm track with an anomalous cyclonic circulation, further amplified by enhanced Kuroshio latent heat release. Our findings suggest that accounting for decadal shifts in KE SST boundary forcing is critical for accurately representing KE downstream impacts.

## Teaser

Two contrasting Kuroshio Extension atmospheric circulation patterns arise from distinct atmospheric boundary conditions.

## INTRODUCTION

Midlatitude air-sea interactions are central to the climate system, governing the mean state of surface winds (*1*–*3*), precipitation (*4*, *5*), and extratropical storm tracks (*6*–*8*). These interactions are especially strong over western boundary currents (WBCs) and their eastward extensions, where surface heat flux exhibits both the largest mean value and the largest variance on interannual to decadal time scales across the global oceans (*9*–*11*). In the North Pacific, for instance, the Kuroshio current transports heat poleward from the tropics and releases substantial amounts of heat and moisture to the atmosphere (*12*, *13*), mainly via latent and sensible heat fluxes during winter when cold and dry monsoonal winds promote surface heat loss (*14*, *15*). The importance of these processes extends beyond the mean climate, with emerging evidence showing the role of WBCs in local and remote climate variability and downstream impacts (*16*, *17*).

As a dominant climate feature in the North Pacific, the Kuroshio Extension (KE) exhibits distinct decadal variations in upper-ocean circulation and heat content (*18*–*20*). Such KE decadal variability is often measured by sea surface height (SSH) anomalies averaged over 31°–36°N, 140°–165°E domain (see fig. S1), which effectively captures the key components of the KE dynamical state, including its path length, intensity, latitudinal position, and the strength of the southern recirculation gyre (*18*). A growing body of evidence indicates that KE variability modulates the North Pacific storm track and downstream atmospheric circulation patterns (*21*–*25*), with far-reaching impacts including precipitation changes over the west coast of North America (*26*–*28*) and central tropical Pacific SST variability (*29*, *30*). These atmospheric remote impacts may serve as a delayed negative feedback mechanism on decadal timescales for the KE dynamic system via oceanic baroclinic Rossby wave propagation (*24*, *29*). However, most of the existing evidence is derived from either observational analyses with relatively short time periods (*21*, *23*, *24*) or from modeling studies targeting specific cases (*25*–*27*). This means that the degree of potential variability in KE atmospheric impacts is still largely unknown.

Within the existing body of evidence, contrasting atmospheric circulation patterns to KE variability have been reported. Some results indicate that during positive KE phases, a stronger and northward shifted SST front anchors the North Pacific storm track in a more poleward position (*21*, *22*, *24*), whereas other studies show a southward migration of the storm track response during positive KE phases, when stronger KE downstream eddy activity leads to enhanced latent heat flux (LHF) release (*25*, *26*). As discussed in ref. (*25*), this ambiguity may stem from the fact that different studies emphasize different aspects of the KE oceanic variability—i.e., the meridional SST gradient versus eddy activity. However, in nature these mechanisms likely operate simultaneously, and their relative importance may vary over time, with opposing atmospheric impacts. What makes this problem even more complex is that remote impacts from outside the Kuroshio region, primarily from the tropical Pacific El Niño-Southern Oscillation (ENSO) atmospheric teleconnection, strongly affect extratropical atmospheric circulation (*11*, *31*), yet different methods adopted across studies to filter out these remote influences can alter the results and lead to divergent conclusions (*22*–*26*). This has made it difficult to date to reconcile the contrasting KE atmospheric circulation patterns, as well as to disentangle how the time horizon of interest influences the KE atmospheric impacts.

To address these gaps, it is necessary to understand the distinct role of different aspects of the KE variability in shaping the atmospheric circulation patterns, while accounting for remote influences from the tropical Pacific ENSO teleconnection. In this respect, we show that the contrasting atmospheric circulation patterns linked to KE variability stem from different aspects of the atmospheric boundary conditions, which are driven by regime-dependent KE relationships with local and remote SST and LHF anomalies. The importance of atmospheric boundary condition changes is further corroborated by a suite of model simulations forced with observed SSTs. Our findings suggest that understanding the contrasting atmospheric circulation responses to KE variability arising from non-stationary KE air-sea interactions could help bridge the gap in the existing literature on KE atmospheric circulation patterns (*25*).

## RESULTS

### Non-stationary relationship between KE SSH and Kuroshio-Oyashio extension (KOE) SST/LHF indices

We first examine the relationship between indices of boreal autumn (September to November, SON) KE SSH and subsequent early winter (December–January, DJ) KOE SST and LHF for the period 1977–2022 (see Materials and Methods for definitions of these indices). The SON value of KE SSH is selected to represent the integrated effects of upper ocean temperature variations during its climatological peak season (fig. S1) (*32*, *33*). In addition, we focus on the LHF response, which dominates the total net surface heat flux (NHF) over the KOE domain, particularly when both NHF and LHF exhibit statistically significant responses to KE variability (fig. S2), as discussed further below. The target season of DJ is chosen because the SON KE index has a distinct seasonal relationship with surface turbulent heat fluxes, exhibiting a statistically significant positive relationship with an upward heat flux response during the early winter season (*23*, *32*), with upward heat flux from the ocean to the atmosphere defined as positive throughout this study. This is due to the seasonal intensification of background northwesterly cold and dry monsoonal winds over East Asia, that enhance surface heat loss over the KOE region (*33*) (fig. S3). We therefore focus on using SON for the KE index and DJ as the target season for the atmospheric and oceanic variables, during which surface turbulent heat flux responses are evident, in line with recent studies based on observational satellite-derived products (*32*, *33*).

**Fig. 1.**
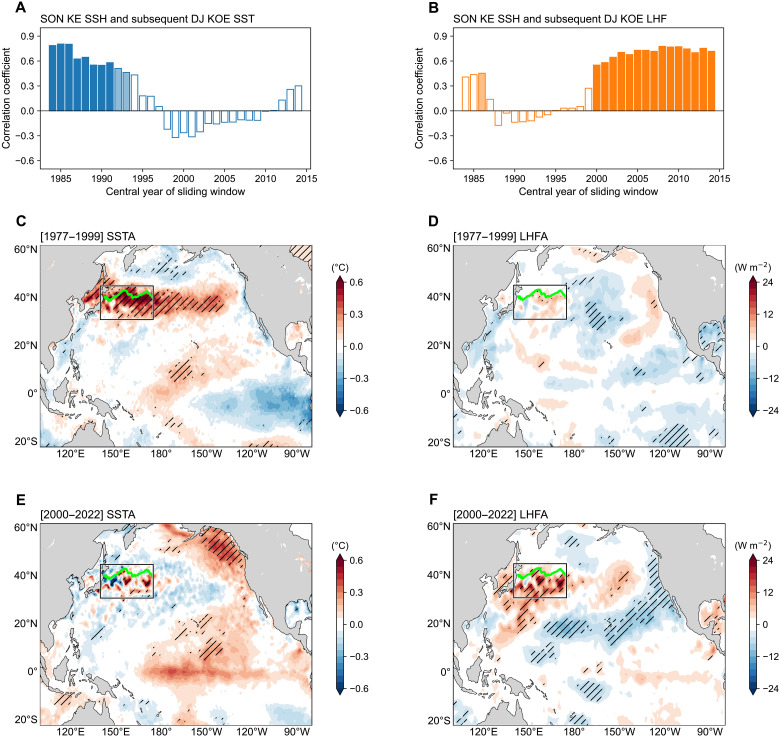
Non-stationary relationship between KE SSH and KOE SST/LHF indices from observational reanalysis. (**A**) The 15-year moving correlation coefficients between SON KE and DJ KOE SST indices during 1977–2022. (**B**) Same as (A), but for DJ KOE LHF index. Bars filled with dark (light) colors indicate significance at the 95% (90%) confidence level, and unfilled bars indicate statistically insignificant relationships. (**C** and **D**) Regression map of DJ (C) SST and (D) LHF anomalies onto the SON KE index during 1977–1999. (**E** and **F**) Same as [(C) and (D)] but for the period 2000–2022. The black box and green line in [(C) to (F)] indicate the KOE domain (31°–45°N, 140°–175°E) and the latitude of maximum climatological meridional SST gradient within the KOE domain (see Materials and Methods), respectively. Hatched areas in [(C) to (F)] indicate regions with regression coefficients statistically significant at the 90% confidence level. In the panel titles, the suffix “A” stands for anomaly.

The 15-year moving correlation analysis reveals that the relationship between the KE SSH index and the KOE SST and LHF indices is non-stationary over the period 1977–2022 (Fig. 1, A and B). The KE SSH index is significantly correlated with the KOE SST prior to the mid-1990s, whereas a significant correlation with the KOE LHF emerges after the late 1990s. A comparison of the correlation coefficients for the period 1977–1999 (hereafter P1) and 2000–2022 (hereafter P2) further highlights this temporal shift in the KE SSH index relationship with the KOE SST and LHF indices (table S1), yet it should be noted that the results remain robust to slight differences in the time windows. These relationship changes are also consistent across multiple independent data sources (fig. S4; see Materials and Methods for dataset details).

During P1 (1977–1999), the KE index has a statistically significant positive relationship with the KOE SST index, but no significant relationship with the KOE LHF index (table S1). The regression map of SST anomalies onto the KE index during P1 shows that positive SST anomalies manifest within the 31°–45°N latitudinal band and extend zonally across the North Pacific (Fig. 1C). A strong climatological meridional SST gradient is found near 41°N within the KOE domain (fig. S5, A and B), corresponding to the subarctic frontal zone (*34*), which is distinct from the strong meridional SSH gradient around 35°N (fig. S5, C and D), referred to as the KE front (*35*). Given its location, the anomalous SST warming associated with the KE index indicates a northward shift of the subarctic SST front (green line in Fig. 1C; see Materials and Methods), consistent with the poleward shift of the KE jet during positive KE phases enhancing warm water advection into the climatological subarctic frontal region (*36*). On the other hand, the regression map of LHF anomalies onto the KE index during P1 shows no statistically significant pattern over the KOE (Fig. 1D). When the LHF response is decomposed into contributions from air-sea humidity difference anomalies (LHF_qdiff_) and surface wind speed anomalies (LHF_wind_) (see Materials and Methods), it is shown that the positive LHF_qdiff_ contribution is offset by the negative LHF_wind_ contribution, yielding a weakly positive but statistically insignificant LHF response during P1 (fig. S6, C and D).

By contrast, the KE index during P2 (2000–2022) has a significant positive relationship with the KOE LHF index, whereas its relationship with the KOE SST index becomes insignificant (table S1). Although we found some statistically significant SST signals within the KOE domain (Fig. 1E), opposing warm and cold anomalies across the KOE region led to an insignificant relationship between the KE SSH and regionally averaged KOE SST indices (Fig. 1A). The SST regression pattern may be linked to the LHF response, with warm (cold) SST anomalies corresponding to upward (downward) LHF anomalies over the KOE region (Fig. 1F). This correspondence in spatial patterns reflects the LHF_qdiff_ contribution (fig. S7, A and C), since warm SST anomalies raise saturation specific humidity and increase air-sea humidity difference, while cold SST anomalies act in the opposite manner. In other words, SST anomalies over the KOE region are damped by surface air-sea heat fluxes, rather than driven by them (*24*, *37*). In addition, the LHF_wind_ term during P2 contributes positively to the LHF response (fig. S7D), in contrast to its negative contribution during P1 (fig. S6D). Taken together, positive contributions from both LHF_qdiff_ and LHF_wind_ give rise to a significant upward KOE LHF response during P2 (Fig. 1B), which can act to dampen the underlying warm SST anomalies and weaken the KE-KOE SST relationship (Fig. 1A).

Beyond the Kuroshio region, we also found anomalous SST warming along with an anomalous negative (i.e., downward) LHF response over the eastern subtropical North Pacific during P2 (Fig. 1, E and F), reminiscent of the wind-evaporation-SST (WES) positive air-sea coupled feedbacks (*38*, *39*), which are less distinct in P1 (Fig. 1, C and D). The warm SST anomaly further extends into the central tropical Pacific region during P2 (Fig. 1E), consistent with the hypothesis that KE downstream atmospheric circulation can influence central tropical Pacific ENSO events through the initiation of WES feedback over the eastern subtropical North Pacific (*29*, *40*). Notably, this SST pattern, with large loadings over the Northeast Pacific and the central equatorial Pacific, resembles the North Pacific-Central Pacific mode (*41*), a decadal timescales dynamical mode of variability that is linked to the development of Northeast Pacific marine heatwaves (*41*, *42*). By comparison, the warm SST anomaly extends only weakly into the central tropical Pacific region during P1, while the eastern tropical Pacific exhibits a cold SST anomaly (Fig. 1C). Recent studies have reported that the strengthened KE SSH and central tropical Pacific SST relationship in recent decades might be associated with changes in KE atmospheric circulation patterns (*40*) and enhanced central tropical Pacific SST variability (*30*). Overall, these results show that KE air-sea interactions exhibit non-stationary changes in their local and remote SST and LHF patterns, with a distinct shift in the LHF_wind_ contribution from negative in P1 to positive in P2 (figs. S6D vs S7D).

### Observed contrasting KE atmospheric circulation patterns

Earlier studies have examined how the KE oceanic heat and moisture source affects extratropical atmospheric circulation, and multiple potential mechanisms have been proposed: (1) the SST front anchoring effect on extratropical storm tracks (*6*, *21*, *24*, *43*) and (2) the midlatitude atmospheric response to latent heating (*23*, *25*, *26*). These mechanisms may depend on whether the atmospheric response arises from the KE-related SST gradient or LHF variations (*23*, *25*). On this basis, we explore KE downstream atmospheric circulation patterns with a focus on the non-stationary relationship between KE SSH and KOE SST/LHF indices.

**Fig. 2.**
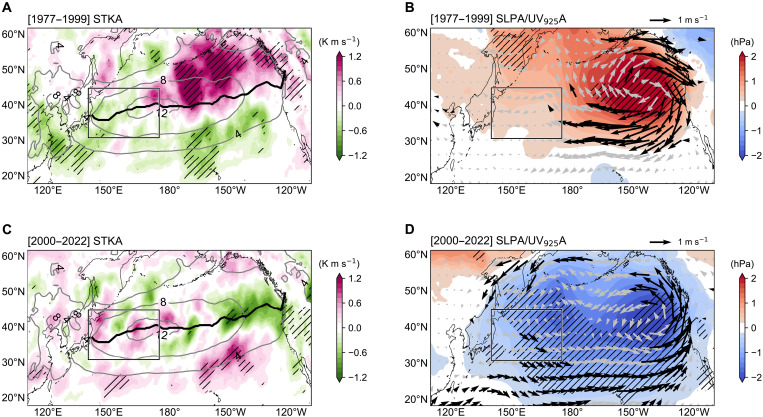
Observed KE atmospheric circulation patterns from observational reanalysis. (**A** and **B**) Regression map of DJ (A) storm track activity (shading) and (B) sea level pressure (SLP; shading) and 925-hPa wind (vector) onto the SON KE index during 1977–1999. (**C** and **D**) same as [(A) and (B)] but for the period 2000–2022. The contours and black line in [(A) and (C)] denote the climatological DJ storm track activity and the latitude of maximum storm track activity at each longitude over the North Pacific, respectively. The black box in [(B) and (D)] denotes the KOE domain. Hatched areas (for shading) and black vectors (for wind) indicate regions where the regression coefficients are statistically significant at the 90% confidence level. In the panel titles, the suffix “A” stands for anomaly.

During P1, the anomalous storm track activity (see Materials and Methods) regressed onto the KE index shows a meridional dipolar pattern along the mean storm track axis with positive anomalies to the northeast and negative anomalies to the southwest over the North Pacific, indicating a poleward migration of the storm track when the KE index is positive (Fig. 2A). This pattern can be interpreted as the SST front anchoring effect, whereby a northward shifted SST front modulates lower-tropospheric baroclinicity, which is estimated by the maximum Eady growth rate at 700-hPa (*44*, *45*) (see Materials and Methods), and anchors extratropical storm tracks in a more poleward position (*6*, *24*) (fig. S8A). In addition, we found anomalous anticyclonic sea level pressure (SLP) patterns over the downstream North Pacific, consistent with previous observational studies (*22*, *46*) (Fig. 2B).

In contrast to P1, we found a southward shift of the downstream North Pacific storm track, albeit with a noisy and weakly organized structure, accompanied by an anomalous cyclonic SLP pattern when the KE index is positive during P2 (Fig. 2, C and D). These circulation anomalies are similar to those associated with an El Niño-like teleconnection driven by warm SST anomalies over the central tropical Pacific (*47*–*49*) (Fig. 1E). Moreover, the enhanced KOE LHF response during P2 leads to stronger local surface evaporation and lower-tropospheric diabatic heating over the KOE region, which is suppressed during P1 when the KOE LHF response is weak (see Materials and Methods; fig. S9). With this heating profile, an anomalous cyclonic circulation is located east of the diabatic heating (Fig. 2D), consistent with the linear midlatitude atmospheric response to thermal forcing (*50*). In this mechanism, the surface low anomaly develops east of the heating such that horizontal temperature advection anomalies act to balance the heating (*51*). However, because the resulting cyclonic circulation strengthens anomalous cold and dry northwesterly winds over the KOE region and further reinforces LHF loss and SLP responses, the observed seasonally regressed pattern alone does not guarantee causality between the LHF and SLP responses (*33*). While an anomalous cyclonic SLP pattern is consistent with the expected linear atmospheric response to local heating, it is likely also affected by remote impacts from central tropical Pacific warm SST anomalies via ENSO teleconnections (*47*–*49*) (Fig. 1E). Hence, we expect that both local and remote forcing contribute to the anomalous cyclonic SLP pattern, which would induce anomalous low-level cold-air advection over the southern flank of the North Pacific. This horizontal temperature advection is consistent with changes in lower-tropospheric baroclinicity (fig. S8B) and a southward shift of the downstream Pacific storm track (Fig. 2C).

### Simulated KE atmospheric circulation patterns

Although we show contrasting KE atmospheric circulation patterns between the late 20th and early 21st centuries, regression analysis by itself cannot establish causality, as atmospheric circulation does also drive SST changes. To address this aspect, we employ a suite of Atmospheric General Circulation Model (AGCM) experiments, consisting of two types of simulations with prescribed observed SSTs. First, we analyze pre-existing transient AGCM simulations using the Community Earth System Model version 2 (CESM2) (*52*) under the “global ocean-global atmosphere” (GOGA) and “tropical ocean-tropical atmosphere” (TOGA) configurations, in which time-varying global and tropical SSTs are prescribed, respectively (see Materials and Methods for experimental details) (*53*, *54*). Each consists of 10 ensemble members, allowing us to isolate the SST-forced response from the contribution of internal atmospheric variability (*55*, *56*).

Building on the insight from the TOGA and GOGA simulations, we then performed a suite of idealized AGCM simulations to assess the atmospheric response to the specific SST pattern associated with the KE index. Using the Department of Energy’s Energy Exascale Earth System Model version 2 (E3SMv2), which contains atmospheric model physics similar to CESM2 (*57*), we ran AGCM experiments forced with SST patterns derived from composite SST anomaly differences between the KE positive and negative phases during P1 and P2 (fig. S10). Given the limitations of the AGCM configuration, in which SST forcing is prescribed under the assumption that the heat capacity of the oceanic mixed layer is infinitely large (*58*), stronger prescribing SST forcing over the KOE region in P1 than in P2 leads to an enhanced upward LHF response (fig. S11). This should be taken into account when interpreting the AGCM results. Nevertheless, we explicitly note that the main purpose of our AGCM simulation is not to reproduce the observed magnitude of the atmospheric response, but rather to capture atmospheric circulation patterns arising from distinct patterns of SST boundary condition in the tropical and North Pacific region between P1 and P2. To investigate this, we conducted a control run with climatological global SSTs and three sensitivity runs in which composite SST anomalies were superimposed on the climatological global SSTs over three regions that exhibit distinct SST anomaly patterns (fig. S10): the tropical Pacific, the North Pacific, and the combined tropical-North Pacific region. The SST-forced atmospheric response in each sensitivity run is estimated as anomalies relative to the corresponding control run for the appropriate period (see Materials and Methods). In the following two subsections, we assess the simulated KE atmospheric circulation patterns for the two periods, P1 (1977–1999) and P2 (2000–2022), in response to the prescribed SST forcing.

#### SST-forced KE atmospheric response during P1 (1977–1999)

**Fig. 3.**
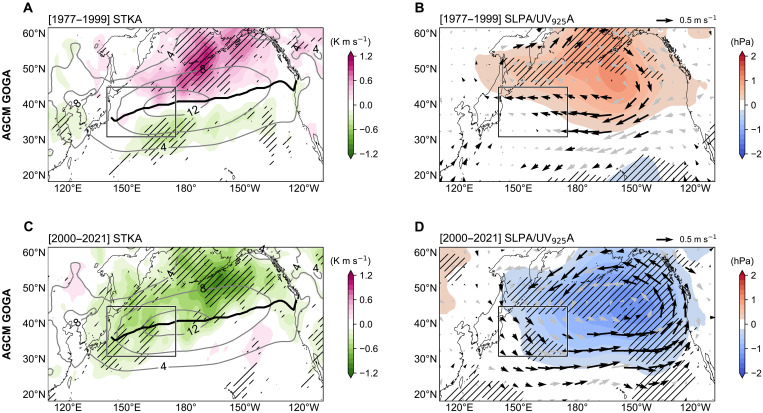
Simulated KE atmospheric circulation patterns from the CESM2 GOGA ensemble. (**A** and **B**) Ensemble-mean regression map of DJ (A) storm track activity (shading) and (B) SLP (shading) and 925-hPa wind (vector) onto the SON KE index during 1977–1999. (**C** and **D**) Same as [(A) and (B)] but for the period 2000–2021. The contours and black line in [(A) and (C)] denote the climatological DJ storm track activity and the latitude of maximum storm track activity at each longitude over the North Pacific, respectively. The black box in [(B) and (D)] denotes the KOE domain. Hatched areas (for shading) and black vectors (for wind) indicate regions where at least 8 out of 10 ensemble members show the same sign in the regression coefficients. In the panel titles, the suffix “A” stands for anomaly.

The TOGA simulation shows only modest ensemble-mean regression patterns of atmospheric circulation onto the KE index during P1 (fig. S12, A and B), with substantial inter-ensemble spread (fig. S13). Despite the moderate observed tropical SST anomaly signals in the SST regression map during P1 (Fig. 1C), the result from the TOGA simulation indicates that the extratropical atmospheric response to tropical SST forcing is weak relative to internal atmospheric variability, and thus cannot account for the observed KE atmospheric circulation. In contrast, GOGA shows a poleward migration of the storm track together with an anomalous anticyclonic SLP pattern over the North Pacific (Fig. 3, A and B), similar to the observed pattern during P1 (Fig. 2, A and B). Moreover, there is robust agreement in the sign of the regression pattern across GOGA ensemble members, as evident in the hatched areas of Fig. 3 where at least 8 out of 10 ensembles agree on the sign (fig. S14 for individual ensemble member regression patterns). With a robust SST-forced response in GOGA but a muted response in TOGA, these results suggest that SST forcing outside the tropics is responsible for the observed anticyclonic circulation pattern and a poleward shift of the storm track linked to KE variability. In particular, the distinct signal of SST anomalies over the KOE region (Fig. 1C) lends further support to this view, in line with the significant KE-KOE SST relationship during P1 (Fig. 1A).

**Fig. 4.**
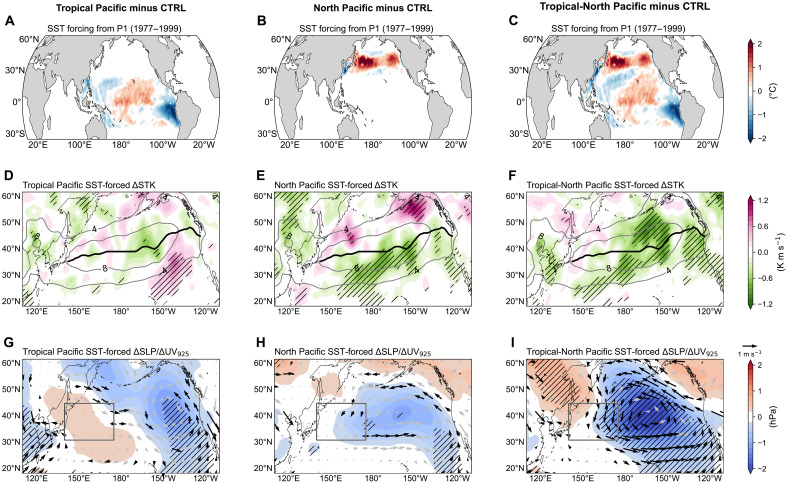
Atmospheric circulation response from AGCM simulations prescribed with KE-related SST forcing derived from P1 (1977–1999). (**A** to **C**), Prescribed DJ SST anomalies from (A) the tropical Pacific, (B) the North Pacific, and (C) the combined tropical-North Pacific simulations for P1 (see Materials and Methods). (**D** to **F**), DJ storm-track activity response from the SST-prescribed runs: (D) the tropical Pacific, (E) the North Pacific, and (F) the combined tropical-North Pacific simulations. (**G** to **I**), Same as [(D) to (F)] but for DJ SLP and 925-hPa wind anomalies. The contours and black line in [(D) to (F)] denote the climatological DJ storm track activity and the latitude of maximum storm track activity at each longitude over the North Pacific, respectively. The black box in [(G) to (I)] denotes the KOE domain. Hatched areas (for shading) and black vectors (for wind) indicate regions where the composite fields are statistically significant at the 90% confidence level. In the panel titles, the Δ symbol denotes anomalies relative to the control simulations.

We then consider the KE SST-forced AGCM simulations derived from P1 (Fig. 4, A to C). The tropical Pacific SST-forced run shows a southward shift of the extratropical storm track with an anomalous cyclonic SLP pattern over the North Pacific (Fig. 4, A, D, and G), reminiscent of an El Niño-like teleconnection pattern (*47*–*49*). When both tropical and North Pacific SST forcing are imposed (Fig. 4C), the North Pacific storm track activity shows an overall weakening (Fig. 4F), while the cyclonic circulation response is amplified relative to the tropical Pacific SST-forced run (Fig. 4I). However, neither of these simulations captures the observed North Pacific storm track and SLP patterns during P1 (Fig. 2, A and B). By contrast, the North Pacific SST-forced run exhibits a poleward migration of the Pacific storm track response (Fig. 4, B and E), similar to that observed in the regression pattern during P1 (Fig. 2A). This result highlights the role of North Pacific SST boundary conditions, whereby a northward shifted SST front acts to anchor the storm track farther poleward, consistent with the SST front anchoring effect (*6*, *43*).

Although the North Pacific SST-forced storm track response during P1 is more consistent with observations and GOGA, its cyclonic SLP response (Fig. 4H) is still at odds with the anticyclonic SLP pattern in observations and GOGA (Fig. 2B and 3B). Given that the GOGA simulation captures the anticyclonic SLP pattern, the full expression of the anticyclonic response may depend on the atmospheric circulation background that is partly set by remote SST boundary conditions (*59*, *60*), including those over the North Atlantic (*61*), which is captured in GOGA but absent in the tropical and North Pacific SST prescribed experiments. Specifically, we identify a statistically significant remote SST signal over the North Atlantic in the regression map of SST anomalies onto the KE index during P1 (30°–50°N, 65°–15°W; black box in fig. S15, A and B). Further analysis shows that North Atlantic SST variability is associated with anticyclonic SLP anomalies over the North Pacific (fig. S15C), with the relationship becoming more pronounced when the North Atlantic SST leads the SLP anomalies by one month (fig. S15D), consistent with evidence from both observations and model experiments (*61*, *62*). Thus, North Atlantic SST forcing may provide a plausible explanation for why the prescribed SST experiments fail to capture the anticyclonic SLP response (Fig. 4H), but GOGA simulation is able to reproduce it (Fig. 3B) by prescribing global SSTs within the same AGCM configuration. Despite these limitations, the North Pacific SST-forced run captures the correct storm track signal: as such, these results strongly indicate that the observed KE storm track pattern during P1 can be largely explained by a robust North Pacific SST-forced response beyond internal atmospheric variability.

#### SST-forced KE atmospheric response during P2 (2000–2022)

The ensemble-mean regression patterns from both TOGA and GOGA can reproduce the observed KE atmospheric circulation patterns during P2, characterized by a southward migration of the storm track and anomalous cyclonic SLP pattern over the North Pacific (Fig. 3, C and D and fig. S12, C and D). The regression fields also exhibit strong agreement across ensemble members (hatched areas in Fig. 3 and fig. S12; see also figs. S16 and S17 for individual ensemble regression patterns), indicating a robust contribution of SST-forced response to the observed KE atmospheric circulation beyond internal atmospheric variability. However, even when identical tropical SST forcing is prescribed, GOGA exhibits a stronger atmospheric response than TOGA. This result suggests that SST forcing outside the tropics may amplify the tropical SST-forced atmospheric response. As discussed in the section “Observed contrasting KE atmospheric circulation patterns,” the observed KE atmospheric circulation during P2 can be interpreted as a remotely driven ENSO teleconnection, a local atmospheric response to KOE thermal forcing, or their combined influence.

**Fig. 5.**
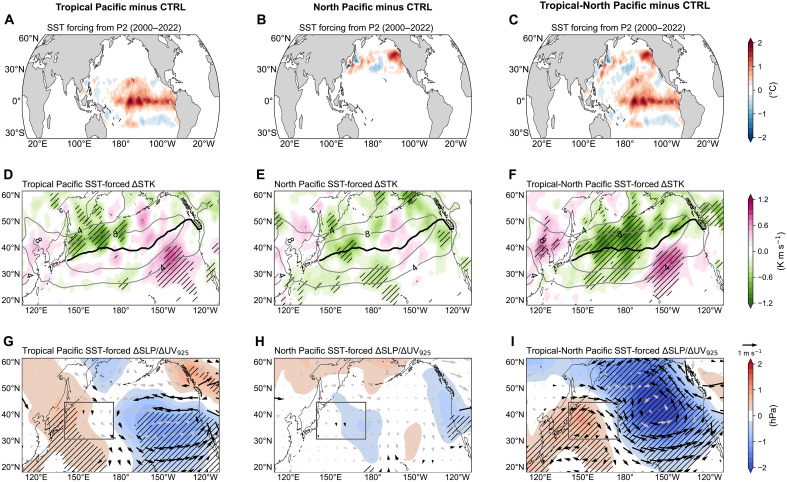
Atmospheric circulation response from AGCM simulations prescribed with KE-related SST forcing derived from P2 (2000–2022). (**A** to **C**) Prescribed DJ SST anomalies from (A) the tropical Pacific, (B) the North Pacific, and (C) the combined tropical-North Pacific simulations for P2 (see Materials and Methods). (**D** to **F**) DJ storm-track activity response from the SST-prescribed runs: (D) the tropical Pacific, (E) the North Pacific, and (F) the combined tropical-North Pacific simulations. (**G** to **I**) Same as [(D) to (F)] but for DJ SLP and 925-hPa wind anomalies. The contours and black line in [(D) to (F)] denote the climatological DJ storm track activity and the latitude of maximum storm track activity at each longitude over the North Pacific, respectively. The black box in [(G) to (I)] denotes the KOE domain. Hatched areas (for shading) and black vectors (for wind) indicate regions where the composite fields are statistically significant at the 90% confidence level. In the panel titles, the Δ symbol denotes anomalies relative to the control simulations.

We then consider the KE-forced AGCM simulations derived from P2 (Fig. 5, A to C): when only the North Pacific SST forcing is imposed, the SST-forced atmospheric circulation response is weak and spatially incoherent (Fig. 5, E and H). Given the weak atmospheric circulation response, the resulting LHF response over the KOE region is primarily driven by the anomalous air-sea humidity difference. As a result, the upward (downward) LHF response is evident over regions of warm (cold) SST forcing (fig. S11E), consistent with increases (decreases) in saturation specific humidity and the air-sea humidity difference. This is further supported by the observed LHF_qdiff_ response and its spatial correspondence with SST anomalies during P2 (fig. S7, A and C). Further, the dominance of warm SST forcing within the KOE domain leads to a net upward LHF response but is insufficient on its own to drive the SLP response over the North Pacific (Fig. 5H). On the other hand, both tropical Pacific and tropical-North Pacific SST forcing runs exhibit a southward migration of the Pacific storm track with an anomalous cyclonic SLP pattern (Fig. 5, D, F, G, and I), similar to that observed regression pattern (Fig. 2, C and D). However, it should be noted that the cyclonic circulation response is stronger in the tropical-North Pacific SST run than in the tropical Pacific SST run, and even exceeds the linear sum of the individual tropical and North Pacific SST runs (fig. 18D). In line with the insights from TOGA and GOGA, these results suggest that the remote effect of tropical Pacific SST forcing can be amplified by the North Pacific SST forcing, with their combined effect being non-linear.

With respect to the non-linear combined effects, we note that the upward LHF response over the North Pacific is substantially amplified in the tropical-North Pacific SST run (fig. S11F), and even larger than the linear sum of the individual tropical and North Pacific SST runs (fig. S18C). The tropical Pacific SST forcing alone drives anomalous cold and dry northwesterly winds over the KOE region (Fig. 5G), yet its influence on the upward LHF response is limited in the absence of underlying warm SST forcing (fig. S11D). In contrast, the LHF response in the tropical-North Pacific SST run is amplified in association with a stronger cyclonic SLP response (Fig. 5I), where underlying warm SST anomalies are prescribed (fig. S11F). Herein, the enhanced LHF loss acts as an atmospheric heat source and may further reinforce the cyclonic SLP response (*33*, *50*), although the causality between the LHF and SLP responses require investigation. Even if the relatively small North Pacific SST forcing during P2 does not fully represent the observed LHF response (fig. S11E) and may underestimate the SLP response (Fig. 5H), we underscore that the impact of North Pacific SST forcing on the atmospheric circulation is amplified when combined with tropical Pacific SST forcing (Fig. 5I; see also fig. S18D).

Such an amplified KOE LHF response is consistent with the fact that the North Pacific SST forcing prescribed in GOGA leads to a stronger upward KOE LHF response than in TOGA (fig. S19). As an atmospheric heat source, the amplified LHF response acts as a boundary forcing that intensifies the cyclonic SLP response via a linear midlatitude atmospheric response to heating (*50*), as seen in GOGA (Fig. 3D). These results suggest that although the North Pacific SST forcing alone exerts only a modest influence on the atmospheric circulation, it acts to nonlinearly amplify the LHF and atmospheric response when combined with the tropical Pacific SST forcing. Thus, the combined effects of tropical and North Pacific SST forcings likely contribute to the observed KE atmospheric circulation pattern during P2. Meanwhile, we note that remote SST forcing outside the Pacific basin may also modulate the atmospheric circulation response. Indeed, the KE index during P2 is negatively correlated with the North Atlantic SST (fig. S20, A and B). Given its potential impact on North Pacific atmospheric circulation (fig. S20, C and D), the remote North Atlantic SST forcing may also contribute to the observed cyclonic SLP response during P2. These results may help explain why GOGA exhibits a stronger cyclonic SLP response than TOGA during P2, in addition to the combined effects of tropical and North Pacific SST forcings.

### Implications for downstream hydroclimate impacts

The influence of KE variability on atmospheric circulation and its downstream hydroclimate impacts has emerged with growing evidence from high-resolution observations and models (*26*–*28*). These studies underscore the importance of fine-scale air-sea interactions in accurately representing KE atmospheric circulation, thereby improving the prediction of downstream hydroclimate impacts over the west coast of North America. In addition to this focus, here we suggest that accounting for decadal shifts in KE atmospheric circulation patterns is equally important for accurately simulating KE downstream impacts.

**Fig. 6.**
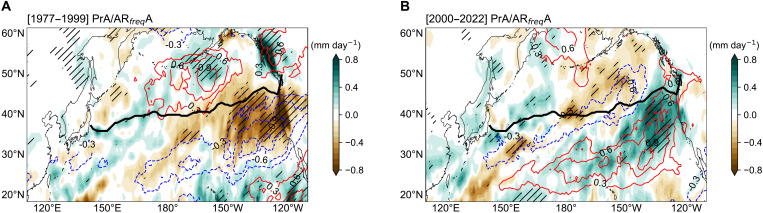
Observed KE downstream hydroclimate impacts from observational reanalysis. (**A** and **B**) Regression map of DJ precipitation (shading, mm day^−1^) and atmospheric river (AR) frequency (red/blue contours, day month^−1^) onto the SON KE index during (A) 1977–1999 and (B) 2000–2022, respectively. The black line denotes the latitude of maximum storm track activity at each longitude over the North Pacific. Hatched areas for shading indicate regions where the regression coefficients are statistically significant at the 90% confidence level. In the figure titles, the suffix “A” stands for anomaly.

In this context, we show that the KE downstream hydroclimate impacts are markedly different before and after the late 1990s. In the earlier decades, the meridional dipole pattern in precipitation and atmospheric river (AR) frequency is evident over western North America (Fig. 6A; see Materials and Methods), reduced in the southern part (i.e., California) and enhanced in the northern part of that region, consistent with the poleward shift of the North Pacific storm track response during P1 (Fig. 2A), whereas in the later decades, increased precipitation and AR frequency are observed over California (Fig. 6B), along the anomalous southwesterly flow of the cyclonic SLP pattern (Fig. 2D). Such observed precipitation patterns are robustly reproduced in the GOGA and North Pacific SST run during P1 (fig. S21, A and C), and in the GOGA and combined tropical-North Pacific SST run during P2 (fig. S21, B and D). These results imply that downstream precipitation responses can differ between different decades even for the same phase of KE variability, depending on the non-stationary nature of KE air-sea interactions and their atmospheric impacts.

## DISCUSSION

Based on observational reanalyses combined with a suite of AGCM simulations, we find that the contrasting KE atmospheric circulation patterns over the past 50 years reflect different aspects of the KE boundary forcing on the atmosphere. Specifically, during the late 20th century (1977–1999), the KE variability exhibited a significant relationship with the KOE SST. During this period, North Pacific SST forcing induces a stronger and northward shifted SST front, which anchors the North Pacific storm track further poleward by modulating atmospheric baroclinicity. With the poleward migration of the North Pacific storm track activity response, reduced precipitation and less frequent AR events are found over California, while enhanced precipitation and more frequent AR events are observed over northern western North America. The GOGA simulation is consistent with this framework: it shows a robust SST-forced response beyond internal atmospheric variability, and the potential role of North Pacific SST forcing is further supported by targeted AGCM sensitivity experiments with specified SST patterns.

In contrast, during the early 21st century (2000–2022), the KE variability showed a stronger connection to the central tropical Pacific SST along with a significant relationship with the KOE LHF. Over this period, the remote effect of tropical Pacific SST forcing induced an anomalous cyclonic SLP pattern and a southward shift of the North Pacific storm track, which was further amplified by the North Pacific SST forcing. This combined effect of the tropical and North Pacific SST forcings is robustly reproduced in the TOGA and GOGA simulations, and targeted sensitivity AGCM experiments further elucidate the nonlinearity in the combined effect. With the anomalous cyclonic flow over the North Pacific, California features enhanced precipitation and more frequent AR events during the positive KE phase, in contrast to the late 20th century. Although the targeted AGCM experiments with specified SST patterns highlight the role of SST forcing within the Pacific basin, we also note the potential contribution of North Atlantic SST forcing as part of the distinct SST boundary conditions that may help explain the contrasting KE-related atmospheric circulation patterns (fig. S15 and S20). These results are in line with previous studies suggesting that remote North Atlantic SST forcing and tropical-extratropical two-way interactions play potentially important roles in shaping the atmospheric circulation over the North Pacific (*29*, *40*, *61*, *63*).

Our findings indicate that the contrasting North Pacific atmospheric circulation responses to KE variability arise from regime-dependent differences in the atmospheric boundary forcing associated with the KE, providing a physical framework for reconciling discrepancies among previous studies, an issue raised in ref. (*25*). Previous studies (*21*, *22*, *24*) focusing on the KE atmospheric circulation primarily prior to 2000 and SST front anchoring mechanisms report results similar to those during P1 (Fig. 2, A and B), whereas post-2000 modeling (*25*–*28*) and observational studies (*23*) focusing on latent heating-induced atmospheric responses show patterns similar to those during P2 (Fig. 2, C and D). These results suggest that accounting for the distinct KE-related SST boundary conditions is important for accurately representing KE atmospheric circulation patterns, with implications for improving predictions of downstream hydroclimate impacts.

An important unresolved question concerns what drives the transitions between these distinct decadal changes. In this respect, we note that the SSH regression maps onto the KE index exhibit a distinct difference in spatial structure between the two periods. During P1, the basin-scale SSH anomalies extend zonally from the KE region across the eastern North Pacific (fig. S22A), reflecting gyre-scale dynamic adjustment of the KE system associated with large-scale wind-forced oceanic Rossby waves (*24*, *64*). As such, the KE jet becomes intensified and northward-shifted, accompanied by a strengthened southern recirculation gyre and broader subtropical gyre circulation, leading to enhanced upper-ocean heat content over a broad region, where warm SST anomalies are broadly collocated with the positive SSH anomalies (Fig. 1C) (*24*, *32*). During P2, SSH anomalies become more localized and confined to the KE domain (fig. S22B), accompanied by an oceanic eddy-like SST pattern (Fig. 1E), indicating a weakened KE SSH-KOE SST relationship in the later period (Fig. 1A). This may be associated with an enhanced KE SSH-KOE LHF relationship that acts to damp the underlying SST anomalies (Fig. 1B). Thus, further analysis of the upper-ocean heat budget would provide insight into differences in SST boundary conditions and help assess the physical processes underlying decadal transitions in KE atmospheric circulation patterns. Further, this question should be addressed in terms of whether they arise from natural climate variability, anthropogenic climate change, or their combined effects. Recent studies have also reported a regime shift in the KE dynamical system after 2018 (*65*), while the KE-related atmospheric circulation patterns remain consistent even when the analysis period is confined to 2017 (fig. S23). These results suggest that different factors may be involved in driving the changes in KE-related atmospheric circulation patterns across the late 1990s, which therefore warrants further investigation.

## MATERIALS AND METHODS

### Dataset

This study primarily uses observational atmospheric and oceanic reanalyses spanning 1977–2022. The monthly SSH is obtained from the European Centre for Medium-Range Weather Forecasts (ECMWF) Ocean Reanalysis System 5 (ORAS5), which assimilates SST, subsurface temperature and salinity profiles at 0.25° horizontal resolution (*66*). Other variables are taken from the ECMWF atmospheric reanalysis (ERA5) with 1° horizontal resolution (*67*): this includes SST, surface latent heat fluxes, sea level pressure, temperature, winds, and precipitation at monthly resolution, as well as daily variables, such as the 850-hPa winds and temperature. To assess the robustness of the reanalysis-based relationship between KE SSH and KOE SST/LHF, we compare these results with those from the eddy-resolving ocean hindcast simulation and satellite-derived products (fig. S4). In this comparison, we employed the Ocean general circulation model for the Earth Simulator (OFES) version 2 (*68*), and satellite-derived products from the Archiving, Validation, and Interpolating of Satellite Oceanographic Level 4 (AVISO) (*69*), Optimum Interpolation SST (OISST) version 2.1 (*70*), and the third-generation Japanese Ocean Flux Dataset using Remote Sensing Observations version (J-OFURO3) (*71*). All anomalies were computed by subtracting the climatological seasonal cycle and removing the long-term linear trend over the period 1977–2022. The main conclusions are robust to computing anomalies and removing linear trends separately within the P1 and P2 periods.

### Definition of KE SSH and KOE SST/LHF indices

We estimated the KE SSH as the area-averaged SSH anomalies over the KE region (31°–36°N, 140°–165°E) where a strong climatological meridional SSH gradient corresponds to an intense eastward flowing oceanic jet (*24*) (fig. S1A). With its seasonal peak in SON (fig. S1B), the KE index shows a distinct seasonal relationship with surface turbulent heat fluxes, exhibiting a significant upward heat flux response over the KOE region during the early winter season in DJ (*32*, *33*) (fig. S3, E to H). This seasonal lag can be explained by the changes in the background atmospheric circulation patterns, in which the Aleutian low extends southwestward in early winter and promotes surface heat loss over the KOE region by advecting cold and dry air (fig. S3). Taking this into account, we used the normalized KE index during SON and analyzed its lagged relationship with SST, LHF, and atmospheric circulation during DJ. The KOE SST and LHF indices are defined as the area-averaged anomalies of SST and LHF over the KOE region (31°–45°N, 140°–175°E), respectively.

### SST front position

We defined the position of the SST front as the latitude of the maximum meridional SST gradient at each longitude over the KOE domain over the period 1977–2022 (*31*, *72*). Within the KOE domain, the maximum meridional SST gradient is found near 41°N (fig. S5, A and B), referred to as the subarctic frontal zone (*34*).

### LHF decomposition

Based on the bulk formula, the LHF anomalies are decomposed into contributions from air-sea humidity difference anomalies (LHF_qdiff_), surface wind speed anomalies (LHF_wind_), and a nonlinear term (LHF_non_) (*73*), using ERA5 and AGCM simulations, as follows

$$\text{LH}F^{\prime }=\rho {\mathrm{LC}}_{E}\left[\underset{{\text{LHF}}_{\text{qdiff}}}{\underbrace{\bar{U}\left(q_{s}^{\prime }-q_{a}^{\prime }\right)}}+\underset{{\text{LHF}}_{\text{wind}}}{\underbrace{U^{\prime }(\bar{q_{s}}-\bar{q_{a}})}}+\underset{{\text{LHF}}_{\text{non}}}{\underbrace{U^{\prime }\left(q_{s}^{\prime }-q_{a}^{\prime }\right)}}\right]$$ (1)

where ρ is atmospheric density, 𝐿 is the latent heat of vaporization, 𝐶_𝐸_ is the bulk coefficient for humidity, 𝑈 is surface wind speed, 𝑞_𝑠_ is specific humidity at the sea surface, and 𝑞_𝑎_ is specific humidity 2 m above the sea surface. In Eq. (1), the first term on the right-hand side corresponds to LHF_qdiff_, the second term to LHF_wind_, and the third term to LHF_non_. As LHF_non_ is negligible compared with the other two terms, our analysis focuses on LHF_qdiff_ and LHF_wind_.

### Storm track activity

Following previous studies (*6*, *8*), the storm track activity is measured as transient eddy temperature fluxes using ERA5 and AGCM simulations, with synoptic eddies defined using a 2–8 day Butterworth band-pass filter applied to daily 850-hPa wind and temperature data. In addition, the storm track axis is defined as the latitude of maximum storm track activity at each longitude over the North Pacific.

### Maximum Eady growth rate

To quantify the atmospheric baroclinicity, we calculate the maximum Eady growth rate as follows

σBI=0.31 gN θ −(∂θ∂y, ∂θ∂x) (2)

where *g* is gravitational acceleration, *N* is the buoyancy frequency, θ is potential temperature, derived from ERA5 data at 700-hPa. This indicates that stronger lower-tropospheric baroclinicity corresponds to weaker static stability and stronger meridional air temperature gradients (*44*, *45*).

### Diabatic heating

In fig. S9, we estimate the diabatic heating profile as the apparent heat source, calculated as the residual of the thermodynamic energy equation, as follows

$$\frac{\mathrm{dT}}{\mathrm{dt}}=-\vec{V}\cdot \nabla T+{\left(\frac{p}{p_{0}}\right)}^{k}\omega \frac{\text{∂}\theta }{\text{∂}p}+\frac{1}{c_{p}}Q_{1}$$ (3)

where *T*, $\vec{V}$, *p*, and ω are the air temperature, the horizontal wind vector, pressure, and vertical pressure velocity, respectively. ∇ denotes the horizontal gradient, and *p*_0_ and *c*_p_ represent the reference pressure at 1000 hPa and the specific heat of dry air at constant pressure, respectively. Q_1_ represents the diabatic heating within the atmospheric column due to radiative fluxes, latent heat release from net condensation, and turbulent sensible heat flux (*74*).

### AGCM simulations

We introduce a suite of AGCM simulations to explore the role of SST-forced atmospheric response in the observed KE atmospheric circulation patterns. It consists of two types of AGCM simulations: transient historical simulations with time-varying SSTs and radiative forcing, and simulations with a particular SST pattern and fixed radiative forcing.

First, we utilize two pre-existing transient AGCM ensembles run with TOGA and GOGA setups under historical radiative forcing using the Community Earth System Model version 2 (CESM2) Community Atmospheric Model version 6 (CAM6) and Community Land Model version 5 (CLM5) with horizontal resolutions of 1.25° longitude by 0.9° latitude (*52*, *53*). These simulations are provided by the NCAR Climate Variability and Change Working Group (*53*). In the TOGA setup, time-varying tropical SSTs are prescribed between 28°S and 28°N, with a linear interpolation of SSTs between 28° and 35° latitude in both hemispheres, and the climatological seasonal cycle of SSTs during 1880–2019 is imposed elsewhere. In the GOGA setup, time-varying global SSTs are prescribed. Sea ice forcing is specified as the climatological seasonal cycle during 1880–2019 for TOGA and 1880–2021 for GOGA. Each simulation consists of 10 ensemble members generated by small perturbations to the initial air temperature. This experimental setup allows us to isolate the SST-forced atmospheric response by calculating regression patterns for each member and averaging them to suppress internal variability (*55*, *56*).

Second, we employ sensitivity AGCM simulations with fixed radiative forcings representative of 2010 using the Department of Energy’s Energy Exascale Earth System Model version 2 (E3SMv2) atmosphere model (EAMv2) and land model (ELMv2) at ∼1° horizontal resolution, which retains similarities to CESM2 in its atmospheric physics and land model (*52*, *57*). A set of four experiments was designed to isolate the role of SST-forced response in shaping the observed KE atmospheric circulation patterns during P1 and P2, respectively. Each set of experiments consists of one control run and three sensitivity runs. The control run is forced with a repeating climatological seasonal cycle of global SSTs, whereas the sensitivity runs are superimposed with the KE-related SST anomalies during December–January over the tropical Pacific, the North Pacific, and their combined regions, respectively (Figs. 4, A to C and 5, A to C). The tropical Pacific domain spans 25°S–25°N and 100°E–65°W with a linearly tapering buffer zone between 20° and 25° in each hemisphere, and the North Pacific domain spans 25°–50°N and 120°E–115°W with linearly tapering buffer zones at 25°–30°N and 45°–50°N, while the combined tropical–North Pacific domain spans 25°S–50°N and 100°E–65°W with linearly tapering buffer zones at 20°–25°S and 45°–50°N (fig. S10). Sea ice forcing is fixed to the climatological seasonal cycle during 1870–1879. Herein, the imposed KE-related SST anomalies are defined as the composite difference of SST anomalies between KE positive and negative phases (KE index exceeding ±0.75 standard deviation; fig. S10), and the resulting atmospheric response is subsequently divided by 2 to account for the use of the composite difference as the SST forcing. Each control and sensitivity run is initialized from a cold start in the startup way and integrated for 35 years. Because the SST forcing repeats identically each model year, individual years in the continuous AGCM simulation can be treated as largely independent ensemble members (*75*). As the equilibrium response to imposed SST forcing, we used the last 30-year average of each control and sensitivity run, noting that results based on the last 30, 25, and 20 years are nearly identical.

### AR frequency

We analyzed ARs detected from ERA5 integrated vapor transport based on the Tracking Atmospheric Rivers Globally as Elongated Targets (tARget) algorithm (*76*). This algorithm, which has been widely applied in global AR studies, was originally developed from ref. (*77*) and validated with dropsonde observations around the western U.S (*78*). The monthly AR frequency is derived from 6-hourly AR data by counting the number of AR occurrences in each grid cell within each calendar month.

### Significance test

Statistical significance was assessed using a two-tailed Student’s *t*-test with N-2 degrees of freedom, where N is the total number of samples. For the CESM2 TOGA and GOGA simulations, inter-ensemble agreement is considered significant when at least 8 out of 10 ensemble members show the same sign in the regression coefficients.
