# Supplementary material for: Two contrasting atmospheric circulation patterns linked to Kuroshio Extension variability
Source: Sci Adv. 2026 Aug 7;12(32):eaee9489. doi: 10.1126/sciadv.aee9489 (PMC13450319; doi:10.1126/sciadv.aee9489)
Supplement: Supplementary file 1 [file sciadv.aee9489_sm.pdf]

Science Advances
AAAS

Supplementary Materials for

Two contrasting atmospheric circulation patterns linked to Kuroshio Extension variability

Se-Yong Song *et al.*

Corresponding author: Se-Yong Song, sysong@ucsb.edu

Sci. Adv. **12**, eaee9489 (2026)
DOI: 10.1126/sciadv.aee9489

This PDF file includes:

Figs. S1 to S23
Table S1

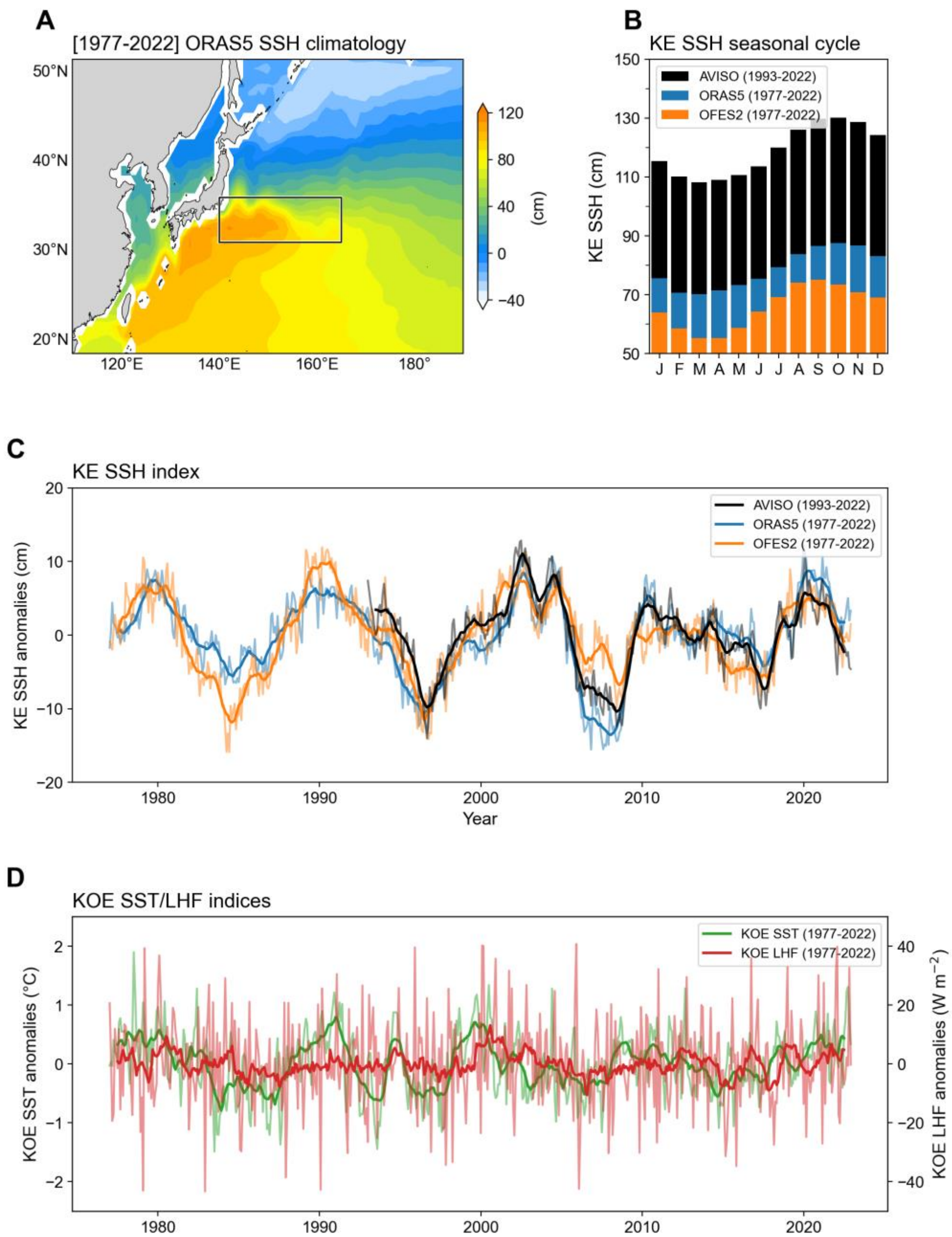

Fig. S1. KE SSH and KOE SST/LHF indices. (**A**) Climatological annual mean SSH patterns during 1977-2022, with the black box indicating the domain used to estimate the KE index (31°-36°N, 140°-165°E). (**B**) Climatological seasonal cycle of KE SSH from different datasets. **(C,D)** Monthly **(C)** KE SSH and (**D**) KOE SST and LHF anomalies, with the 12-month moving average shown as thick colored lines.

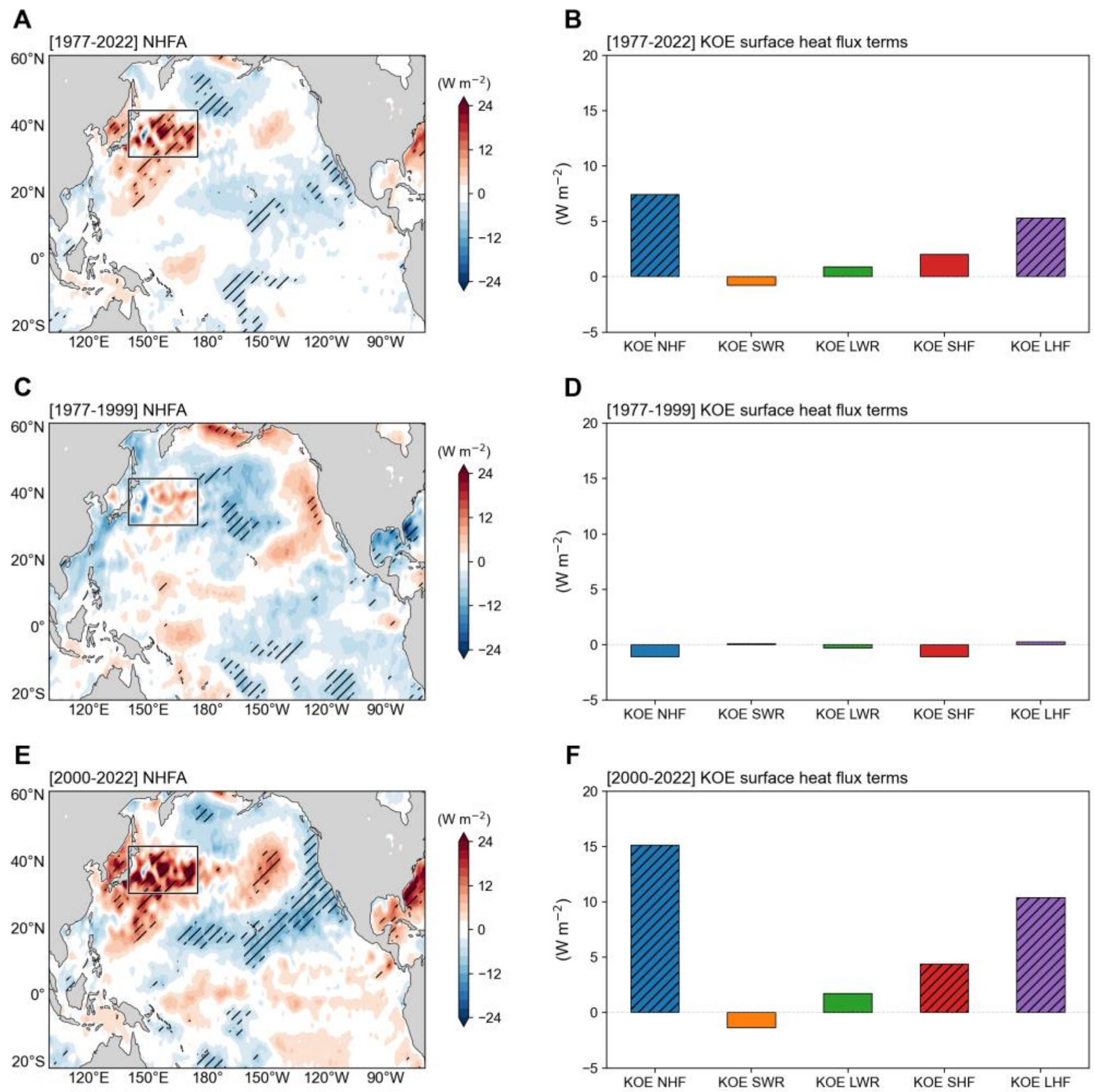

Fig. S2. Surface radiative and turbulent heat flux liked to KE variability. (**A,C,E**) Regression map of DJ surface net heat flux (NHF) anomalies onto the SON KE index during (**A**) 1977-2022, (**C**) 1977-1999, and (**E**) 2000-2022. Here, NHF is defined as the sum of surface net shortwave radiation (SWR), longwave radiation (LWR), sensible heat flux (SHF), and latent heat flux (LHF), where positive values indicate upward heat flux from the ocean. The black box denotes the Kuroshio-Oyashio Extension (KOE) domain (31°-45°N, 140°-175°E). (**B,D,F**) Regression coefficients of KOE area-averaged DJ NHF, SWR, LWR, SHF, and LHF onto the SON KE index during (**B**) 1977-2022, (**D**) 1977-1999, and (**F**) 2000-2022. Hatching indicates values with regression coefficients statistically significant at the 90% confidence level. In the figure titles, the suffix "A" stands for anomaly.

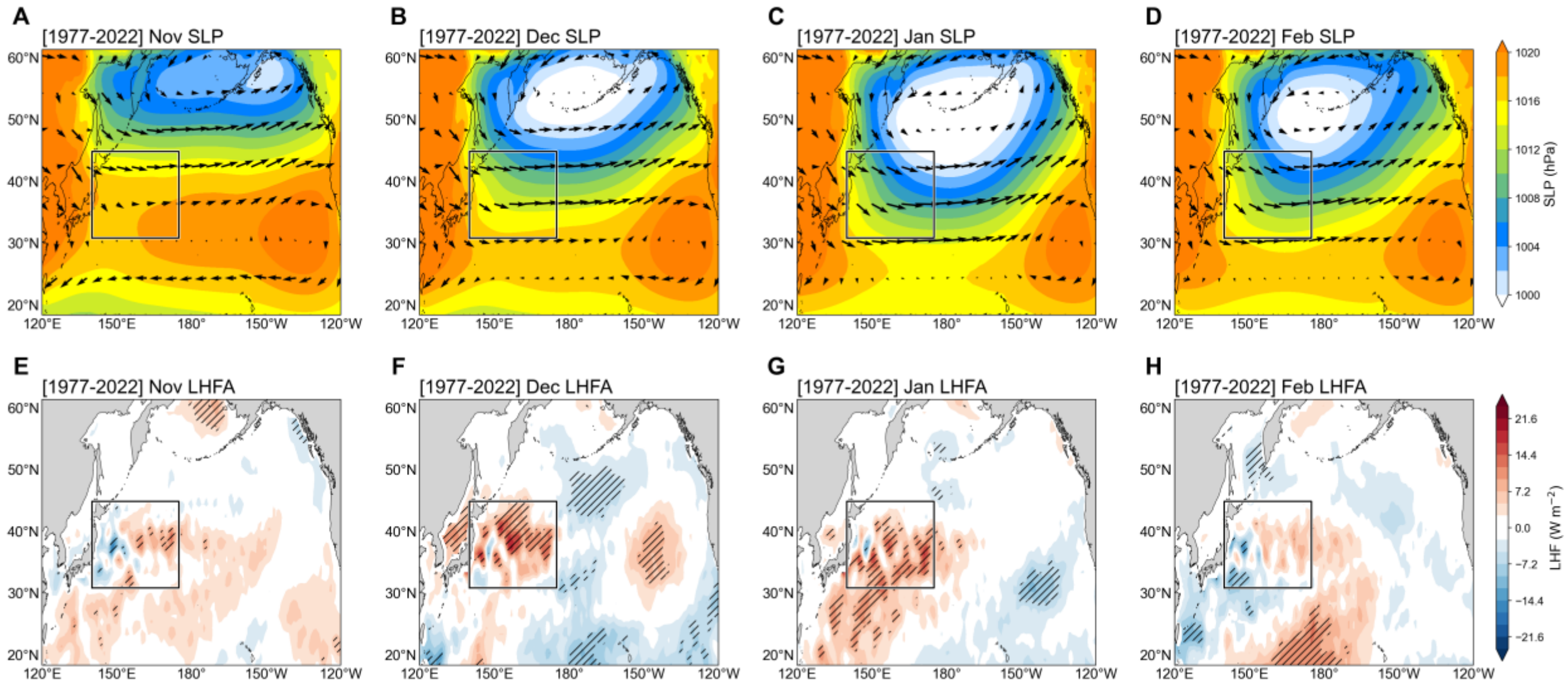

Fig. S3. Climatological atmospheric circulation pattern and regression map of surface latent heat flux (LHF) from November to February during 1977-2022. (**A to D**) Climatological sea level pressure (SLP, shading) and 925-hPa wind (vector) for (**A**) November, (**B**) December, (**C**) January, and (**D**) February, respectively. (**E to H**) Same as (**A to D**), except for the regression maps of monthly LHF onto the SON KE index, where hatching indicates regions with regression coefficients statistically significant at the 90% confidence level. The black box denotes the KOE domain (31°-45°N, 140°-175°E). In the figure titles in (**E to H**), the suffix "A" stands for anomaly.

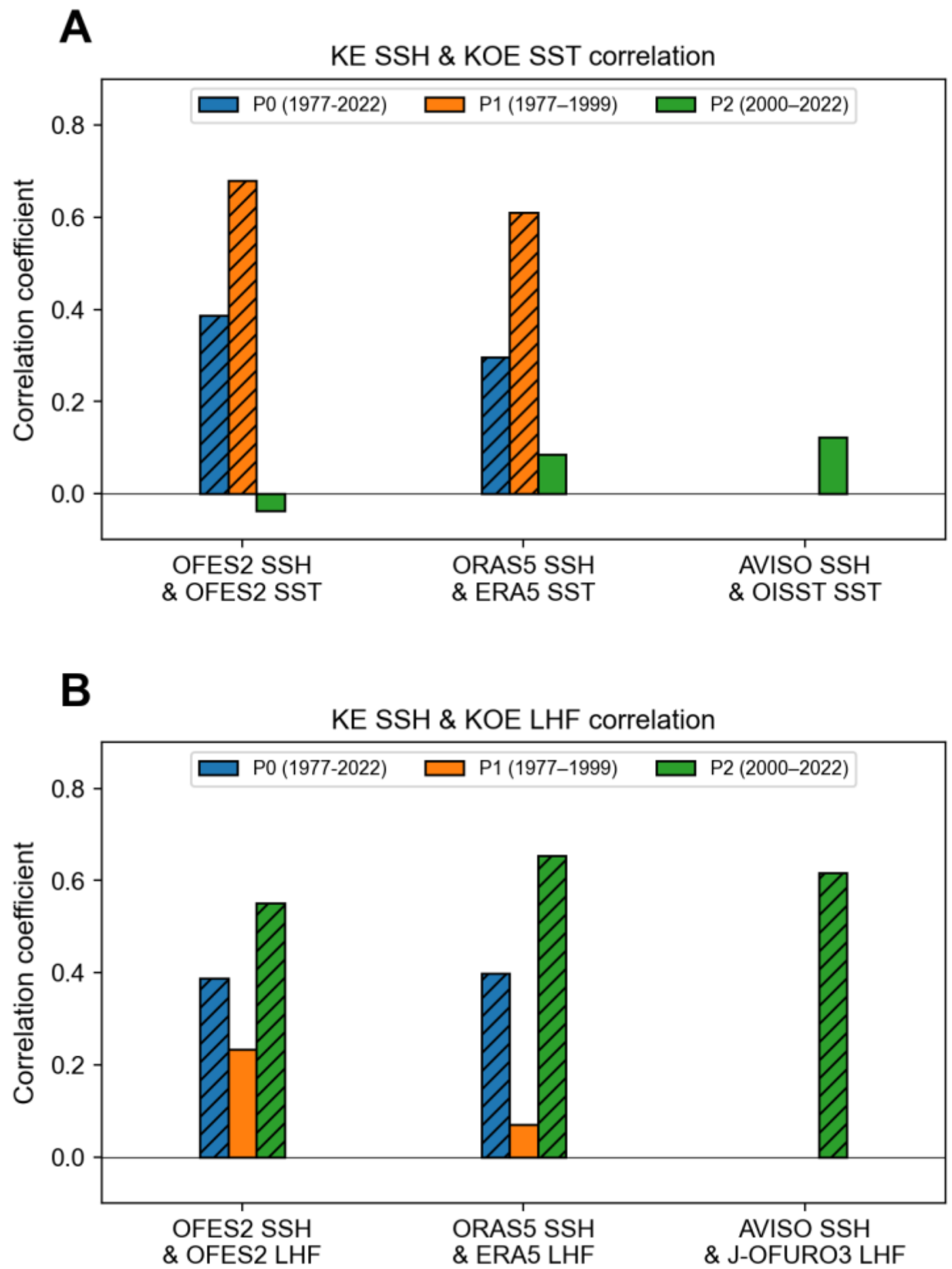

Fig. S4. Changes in the relationship between KE SSH and KOE SST and LHF indices from different datasets. (**A**) The correlation coefficients between SON KE SSH and DJ KOE SST indices during 1977-2022, 1977-1999, and 2000-2022 based on eddy-resolving ocean hindcast simulations (OFES2), reanalysis datasets (ORAS5 and ERA5), and satellite-derived products (AVISO, OISST, and J-OFURO3). (**B**) Same as (**A**) but for the correlation coefficients between KE SSH and KOE LHF indices. Hatching indicates values with correlation coefficients statistically significant at the 90% confidence level.

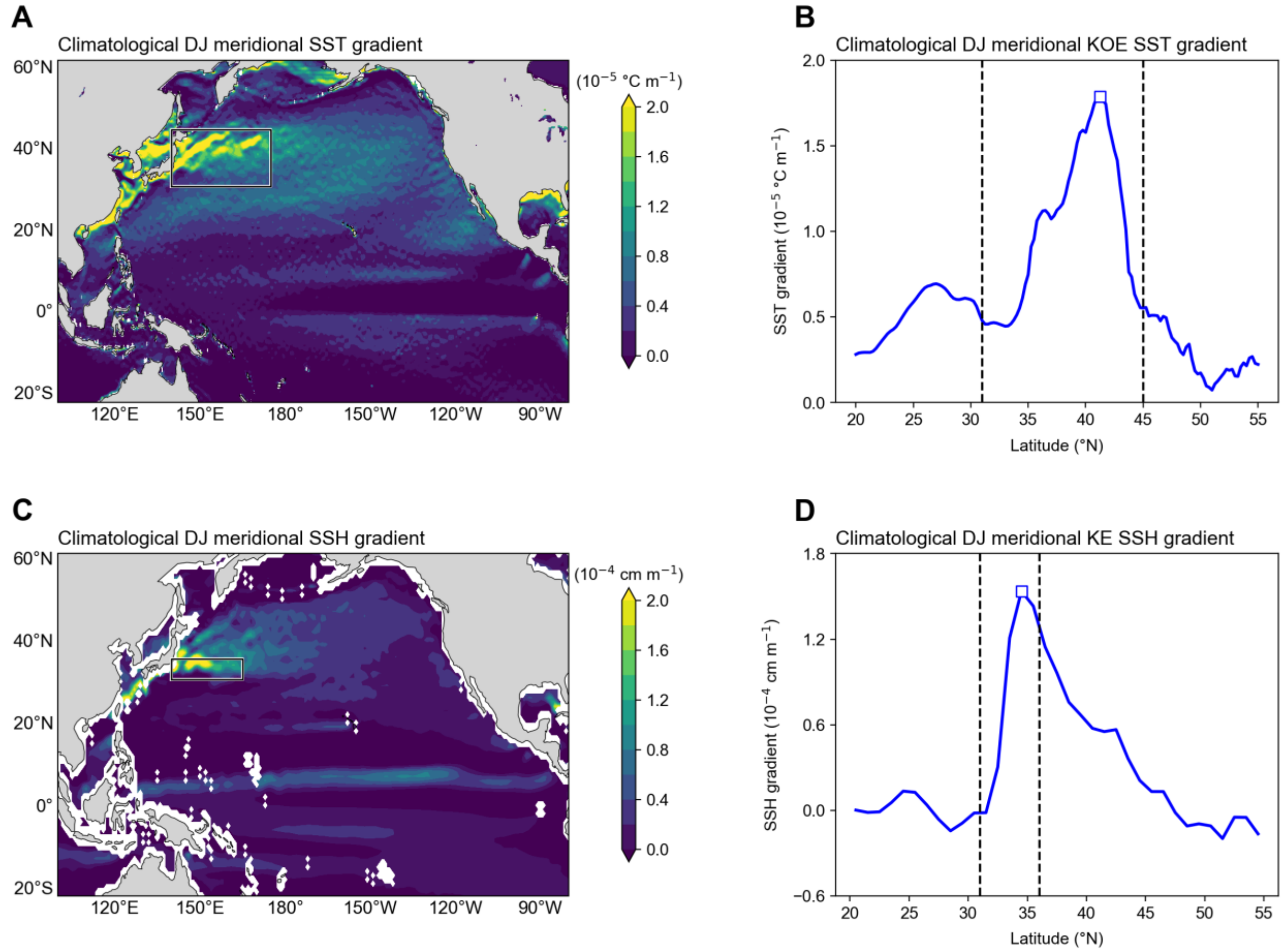

Fig. S5. Climatological meridional SST and SSH gradient. (**A,C**) Climatological DJ meridional (**A**) SSH and (**C**) SST gradient, with the black boxes indicating the KOE (31°-45°N, 140°-175°E) and KE (31°-36°N, 140°-165°E) domains, respectively. (**B,D**) Climatological DJ zonally averaged meridional (**B**) KOE SST and (**D**) KE SSH gradient, with the vertical dashed lines indicating 31°-45°N and 31°-36°N, respectively, and squares marking the latitude of maximum gradient.

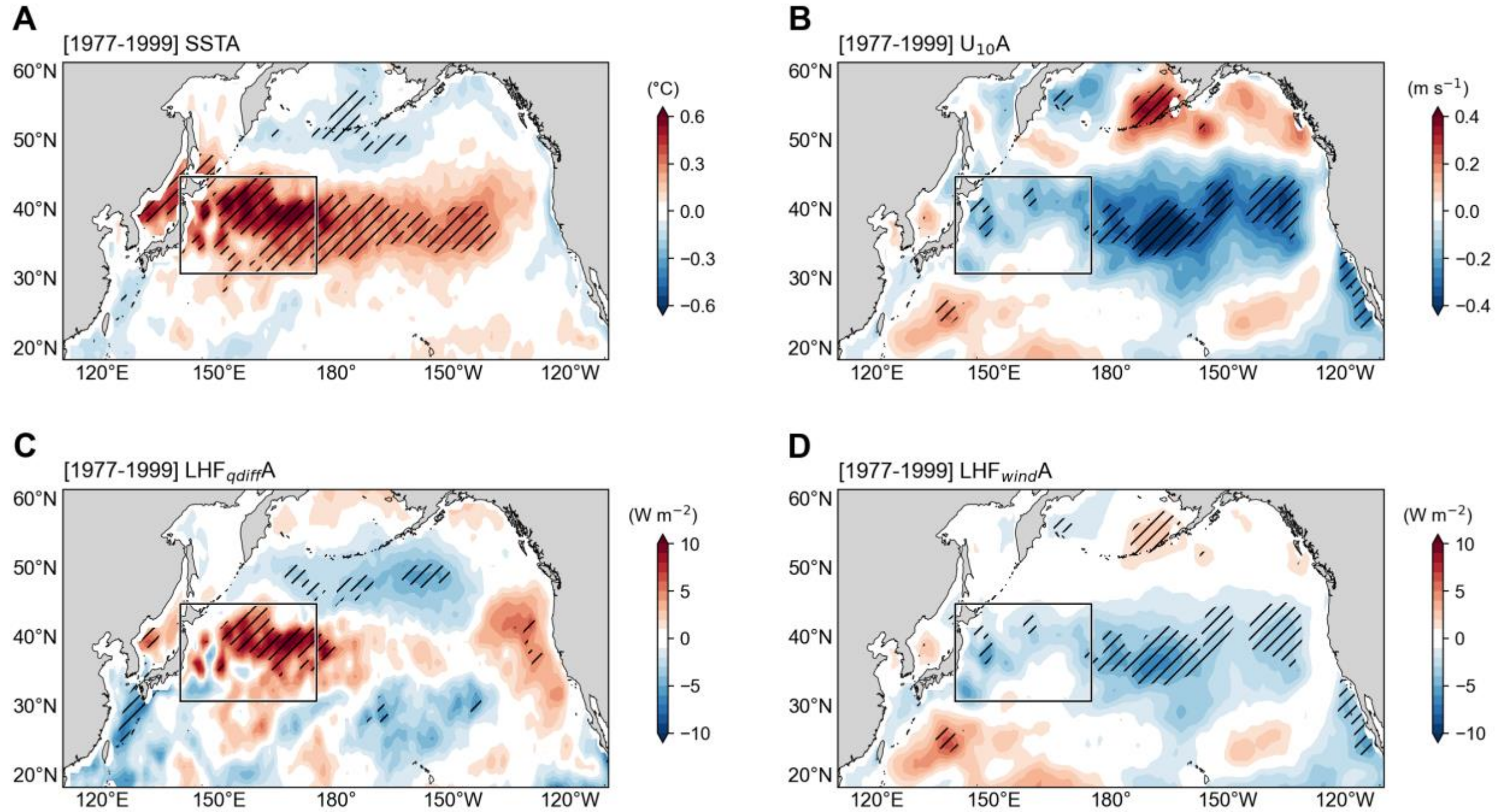

Fig. S6. The SST, surface wind, and decomposed LHF patterns during 1977-1999. (**A to D**) Regression map of DJ (**A**) SST, (**B**) surface wind, (**C**) LHF contribution from air-sea humidity difference anomalies (LHF_{qdiff}), and (**D**) LHF contribution from surface wind anomalies (LHF_{wind}) onto the SON KE index during 1977-1999. The black box denotes the KOE domain. Hatched areas indicate regions with regression coefficients statistically significant at the 90% confidence level. In the figure titles, the suffix "A" stands for anomaly.

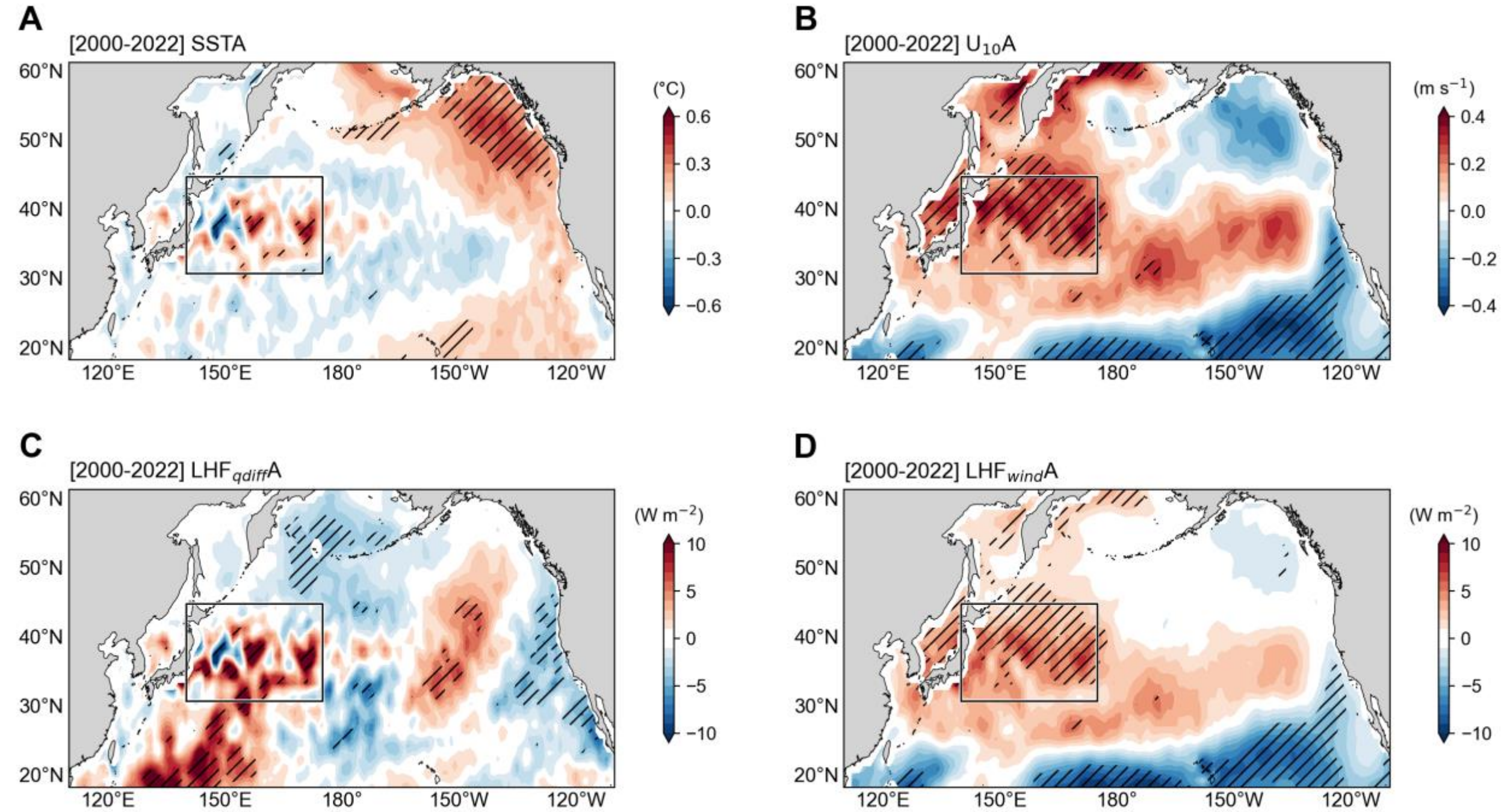

Fig. S7. The SST, surface wind, and decomposed LHF patterns during 2000-2022. Same as fig. S6, but for the period 2000-2022.

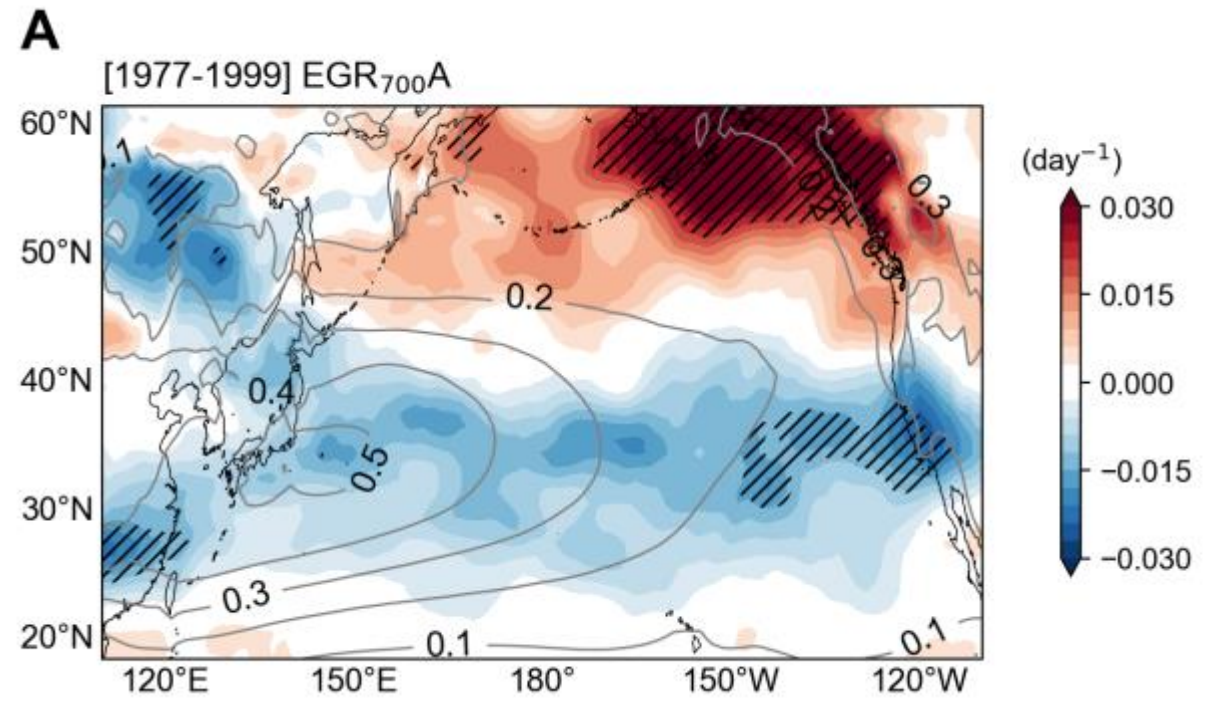

Fig. S8. The maximum Eady growth rate at 700-hPa patterns. (**A,B**) Regression map of DJ 700-hPa maximum Eady growth rate onto the SON KE index during (**A**) 1977-1999 and (**B**) 2000-2022, respectively. The contours denote the climatological DJ maximum Eady growth rate. Hatched areas indicate regions with regression coefficients statistically significant at the 90% confidence level. In the figure titles, the suffix "A" stands for anomaly.

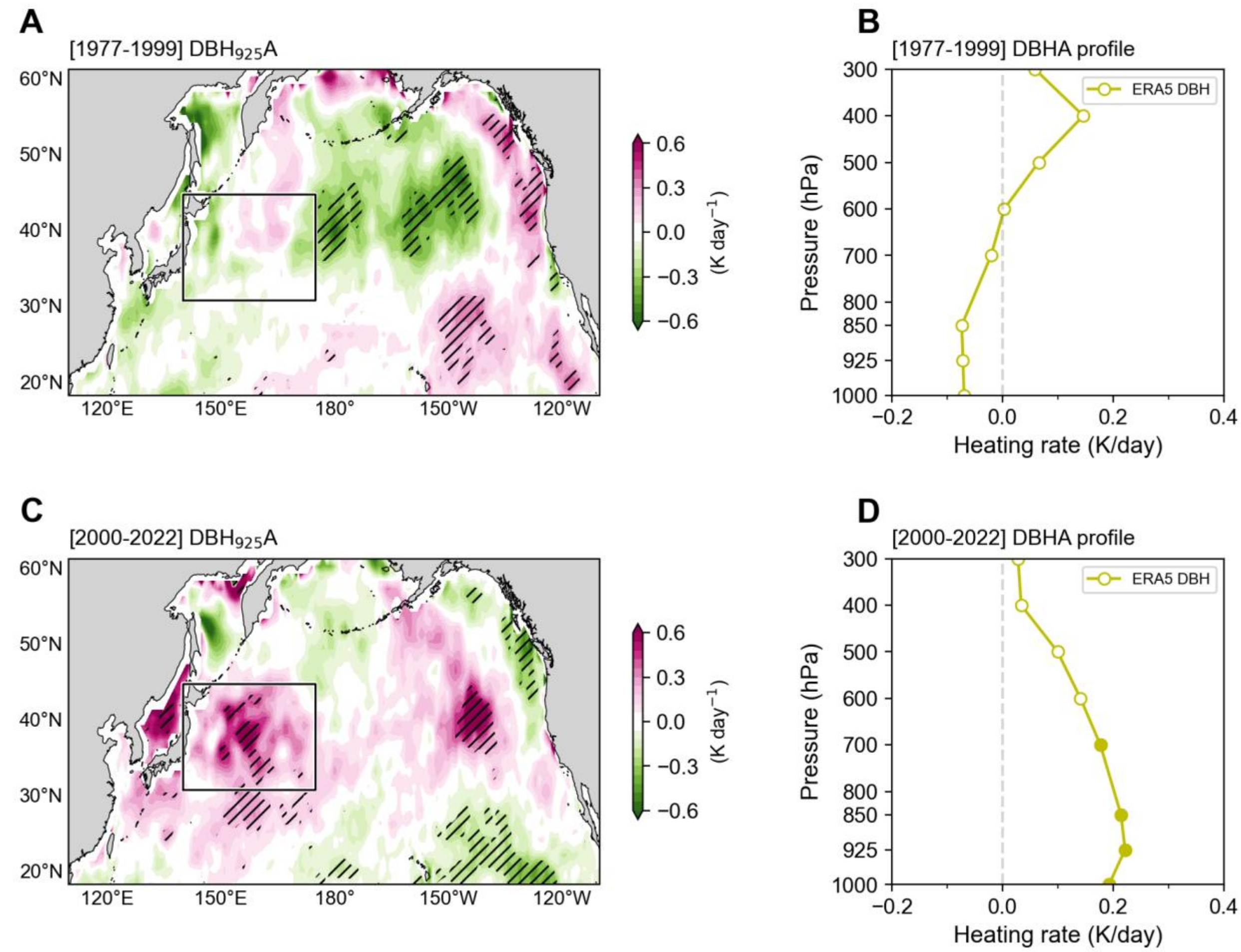

Fig. S9. The diabatic heating at 925-hPa patterns. (**A,C**) Regression map of DJ 925-hPa diabatic heating during (**A**) 1977-1999 and (**C**) 2000-2022, respectively. The black box in (**A,C**) denotes the KOE domain. Hatched areas indicate regions with regression coefficients statistically significant at the 90% confidence level. (**B,D**) Regression profiles of DJ KOE diabatic heating during (**B**) 1977-1999 and (**D**) 2000-2022, respectively, with filled dots indicating values where the regression coefficients are statistically significant at the 90% confidence level. In the figure titles, the suffix “A” stands for anomaly.

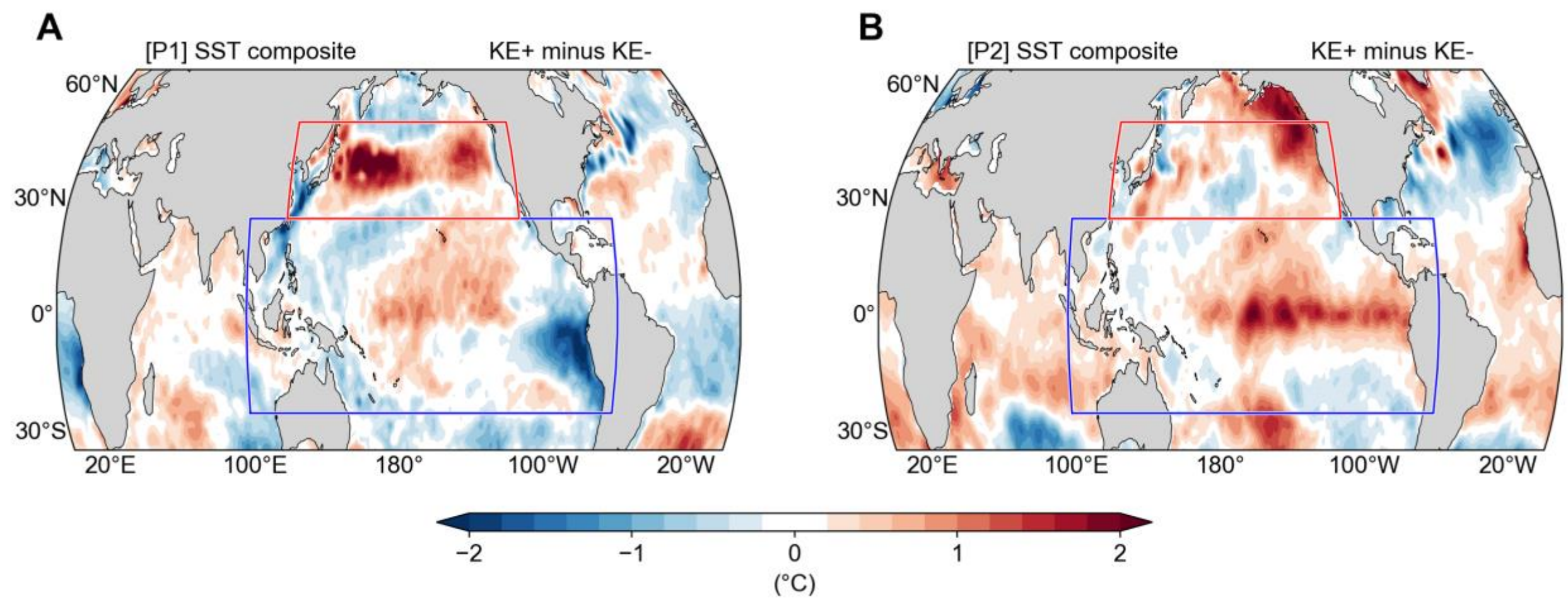

Fig. S10. The SST forcing patterns prescribed in the idealized E3SMv2 AGCM simulation. (**A**) Composite map of SST anomalies based on the difference between KE positive and negative phases (KE index exceeding ±0.75 standard deviation) during P1 (1977-1999). (**B**) Same as (**A**), but for composite map of SST anomalies during P2 (2000-2022). The blue and red boxes denote the tropical Pacific (25°S-25°N, 100°E-65°W) and the North Pacific (25°-50°N, 120°E-115°W) domains, respectively.

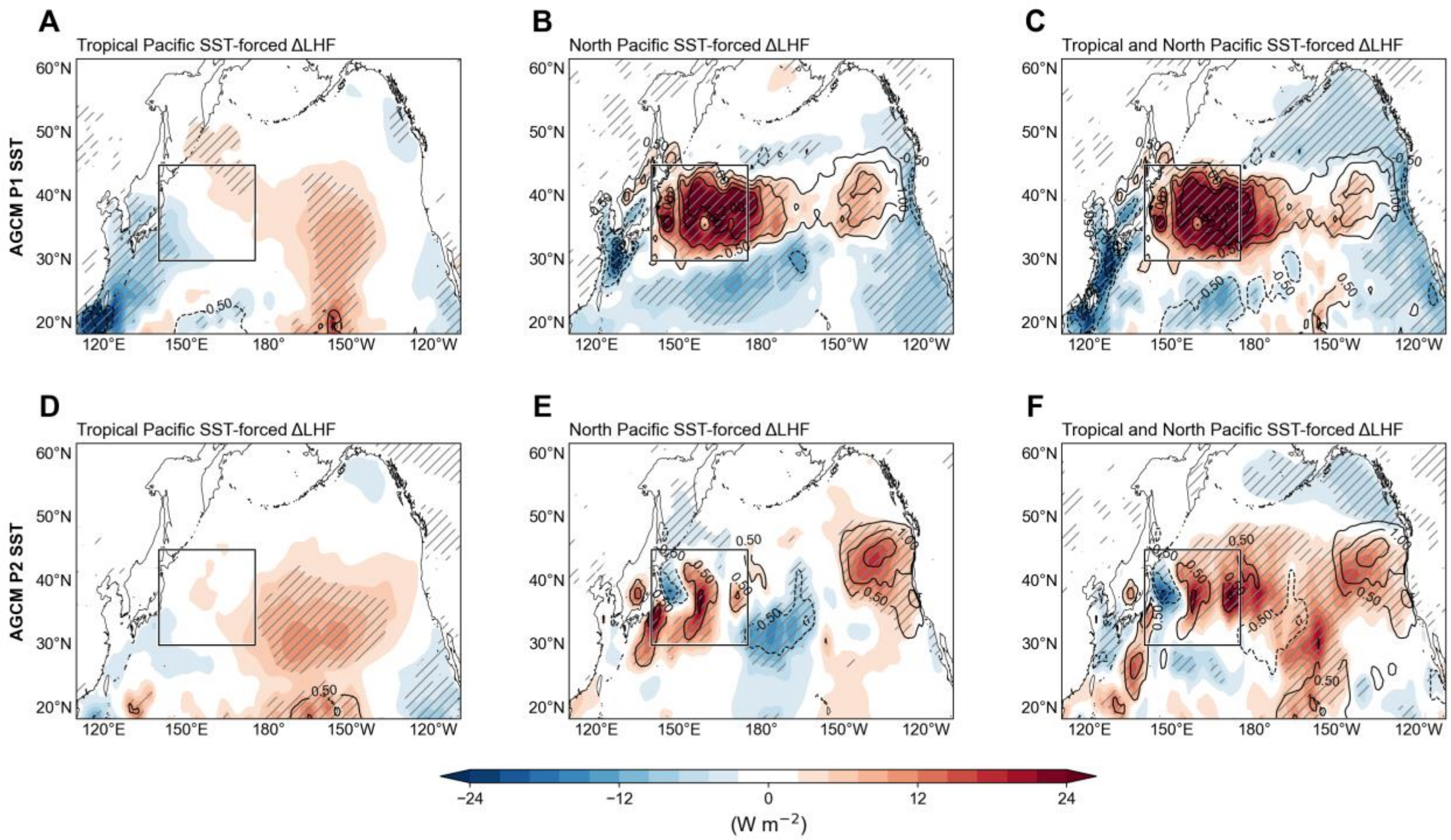

Fig. S11. Surface latent heat flux (LHF) response from E3SMv2 idealized AGCM simulations prescribed with SST patterns derived from P1 (1977-1999) and P2 (2000-2022), respectively. (**A to C**) DJ LHF response (shading, W m^{-2}) and prescribing SST forcing (contour, °C) from the (**A**) Tropical Pacific, (**B**) North Pacific, and (**C**) Tropical and North Pacific SST-forced run for AGCMs forced with SST patterns derived P1. (**D to F**) Same as (**A to C**), but for AGCMs forced with SST patterns derived from P2. Hatched areas indicate regions where the regression coefficients are statistically significant at the 90% confidence level. In the figure titles, the Δ symbol denotes anomalies relative to the control simulations.

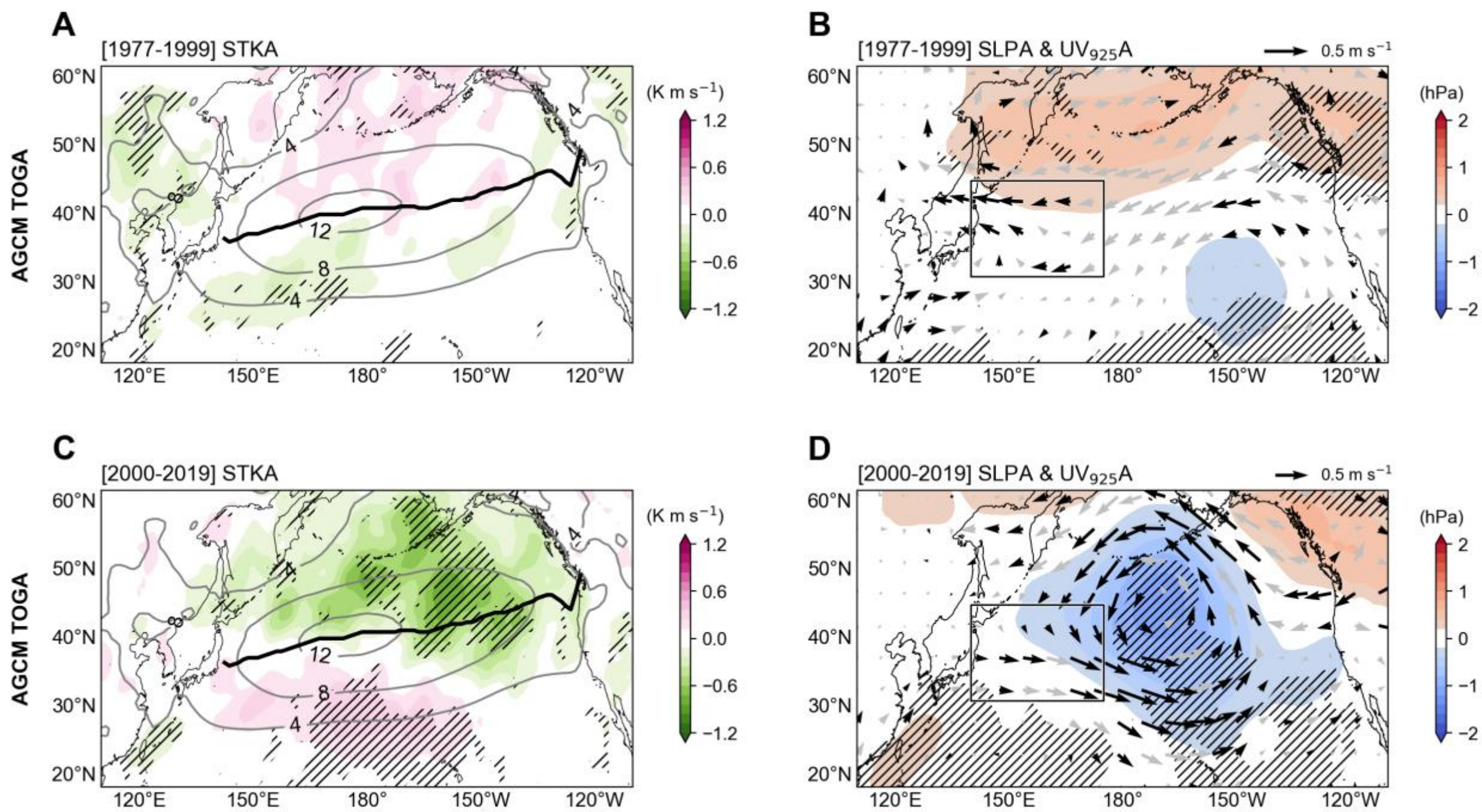

Fig. S12. Simulated KE atmospheric circulation patterns from the CESM2 TOGA ensemble. (**A,B**) Ensemble-mean regression map of DJ (**A**) storm track activity (shading) and (**B**) SLP (shading) and 925-hPa wind (vector) onto the SON KE index during 1977-1999. (**C,D**) Same as (**A,B**) but for the period 2000-2019. The contours and black line in (**A,C**) denote the climatological DJ storm track activity and the latitude of maximum storm track activity at each longitude over the North Pacific, respectively. The black box in (**B,D**) denotes the KOE domain. Hatched areas (for shading) and bold vectors (for wind) indicate regions where at least 8 out of 10 ensemble members show the same sign in the regression coefficients. In the figure titles, the suffix "A" stands for anomaly.

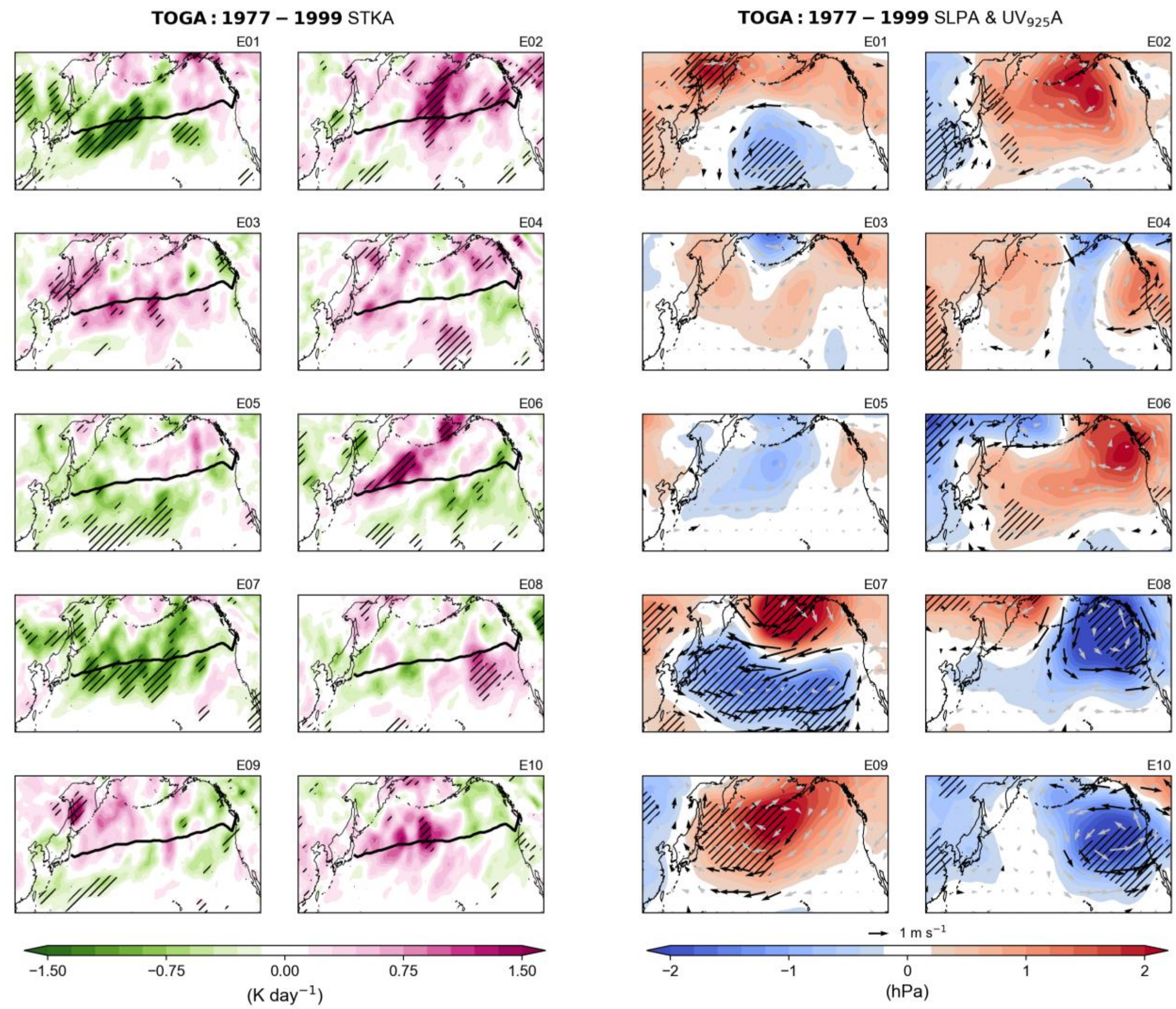

Fig. S13. Simulated KE atmospheric circulation patterns from individual ensemble members in the AGCM TOGA ensemble during 1977-1999. (left) Regression map of DJ storm track activity onto the SON KE index, with the black line indicating the latitude of maximum storm track activity at each longitude over the North Pacific. **(right)** Regression map of DJ SLP (shading) and 925-hPa wind (vector) onto the SON KE index. Hatched areas (for shading) and bold vectors (for wind) indicate regions where at least 8 out of 10 ensemble members show the same sign in the regression coefficients. Panel titles "Exx", where xx ranges from 01 to 10, indicate the ensemble member number used to generate each panel. In the figure titles, the suffix "A" stands for anomaly.

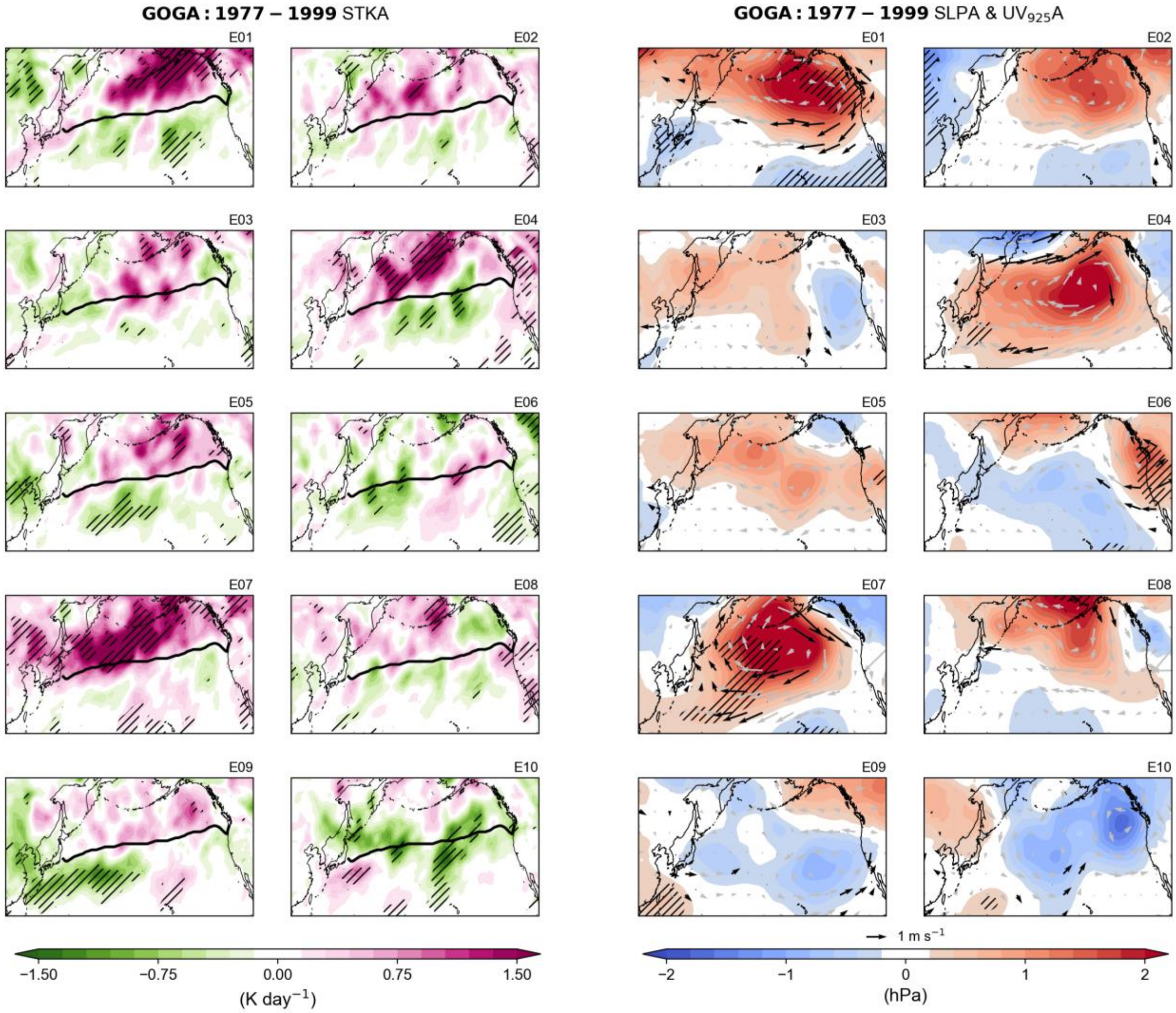

Fig. S14. Simulated KE atmospheric circulation patterns from individual ensemble members in the AGCM GOGA ensemble during 1977-1999. Same as fig. S13, but for the AGCM GOGA ensemble during 1977-1999.

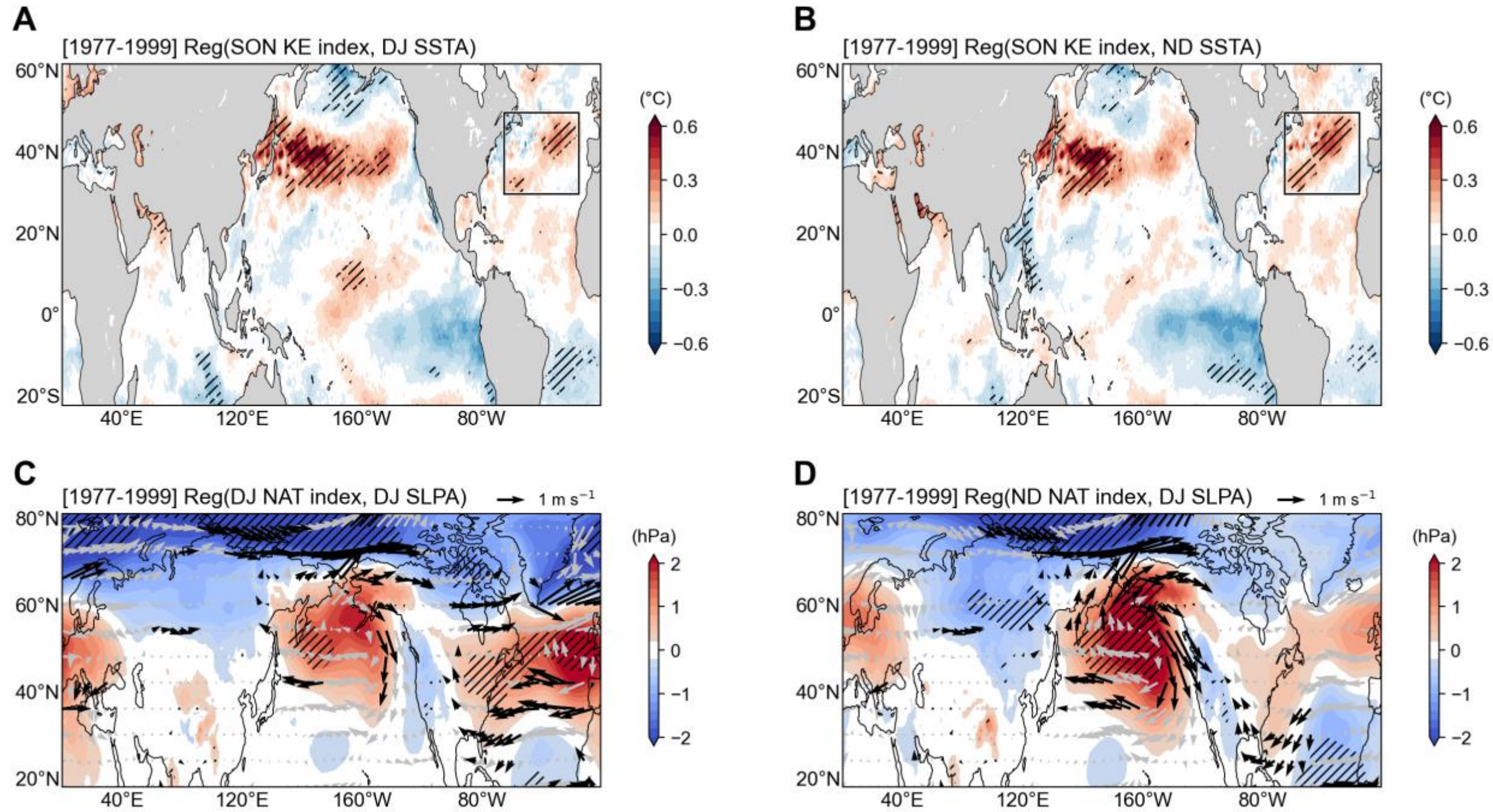

Fig. S15. Remote SST forcing from outside the tropical and North Pacific during 1977-1999. (**A,B**) Regression map of SST anomalies during (**A**) DJ and (**B**) ND onto the SON KE index during 1977-1999. (**C,D**) Regression map of DJ SLP anomalies onto the (**C**) DJ and (**D**) ND North Atlantic SST (NAT) index during 1977-1999. The black box in (**A,B**) denotes the North Atlantic domain (30°-50°N, 65°-15°W) used to define the NAT index. Hatched areas indicate regions with regression coefficients statistically significant at the 90% confidence level. In the figure titles, the suffix "A" stands for anomaly.

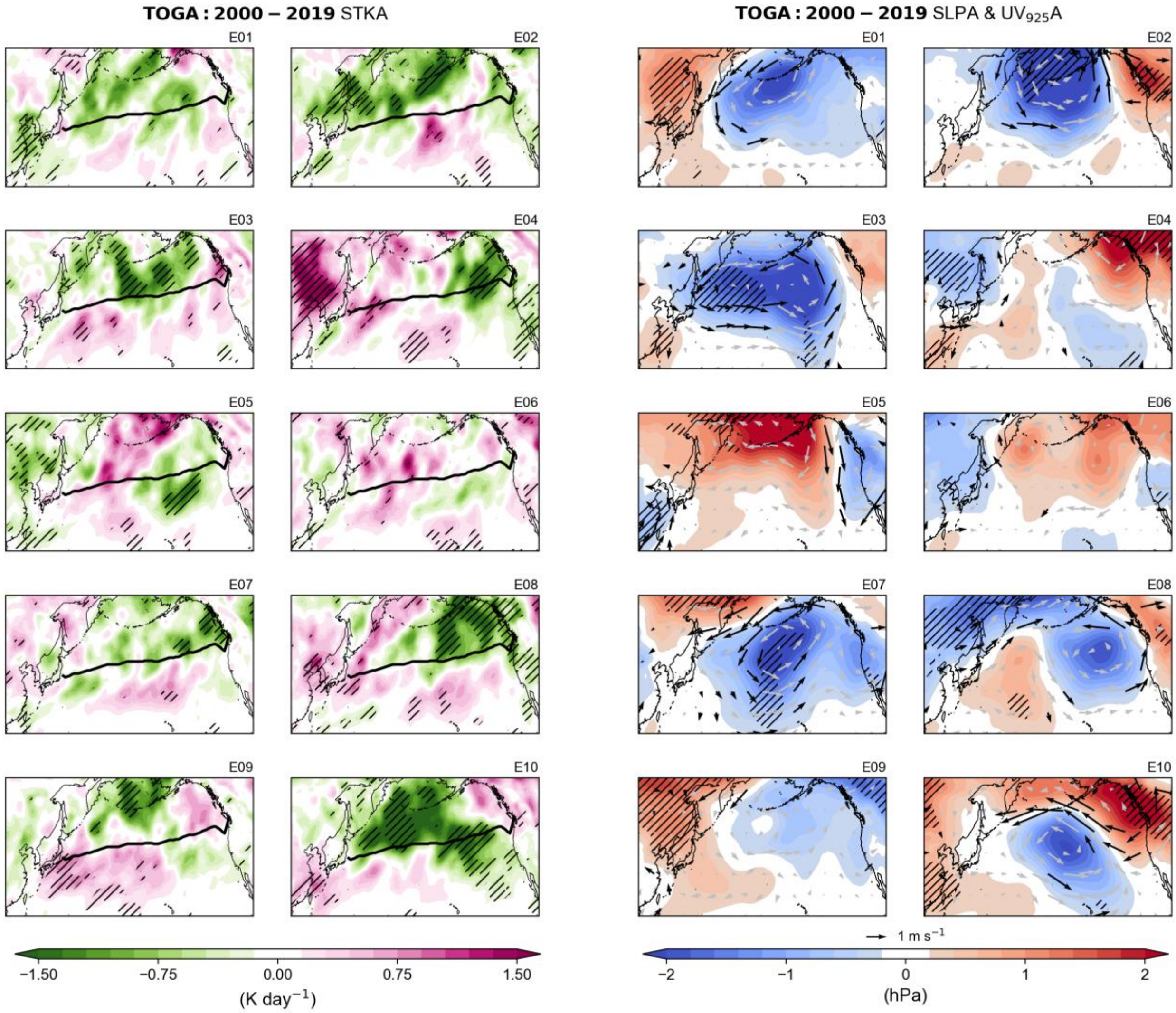

Fig. S16. Simulated KE atmospheric circulation patterns from individual ensemble members in the AGCM TOGA ensemble during 2000-2019. (left) Regression map of DJ storm track activity onto the SON KE index, with the black line indicating the latitude of maximum storm track activity at each longitude over the North Pacific. **(right)** Regression map of DJ SLP (shading) and 925-hPa wind (vector) onto the SON KE index. Hatched areas (for shading) and bold vectors (for wind) indicate regions where the regression coefficients are statistically significant at the 90% confidence level. Panel titles "Exx", where xx ranges from 01 to 10, indicate the ensemble member number used to generate each panel. In the figure titles, the suffix "A" stands for anomaly.

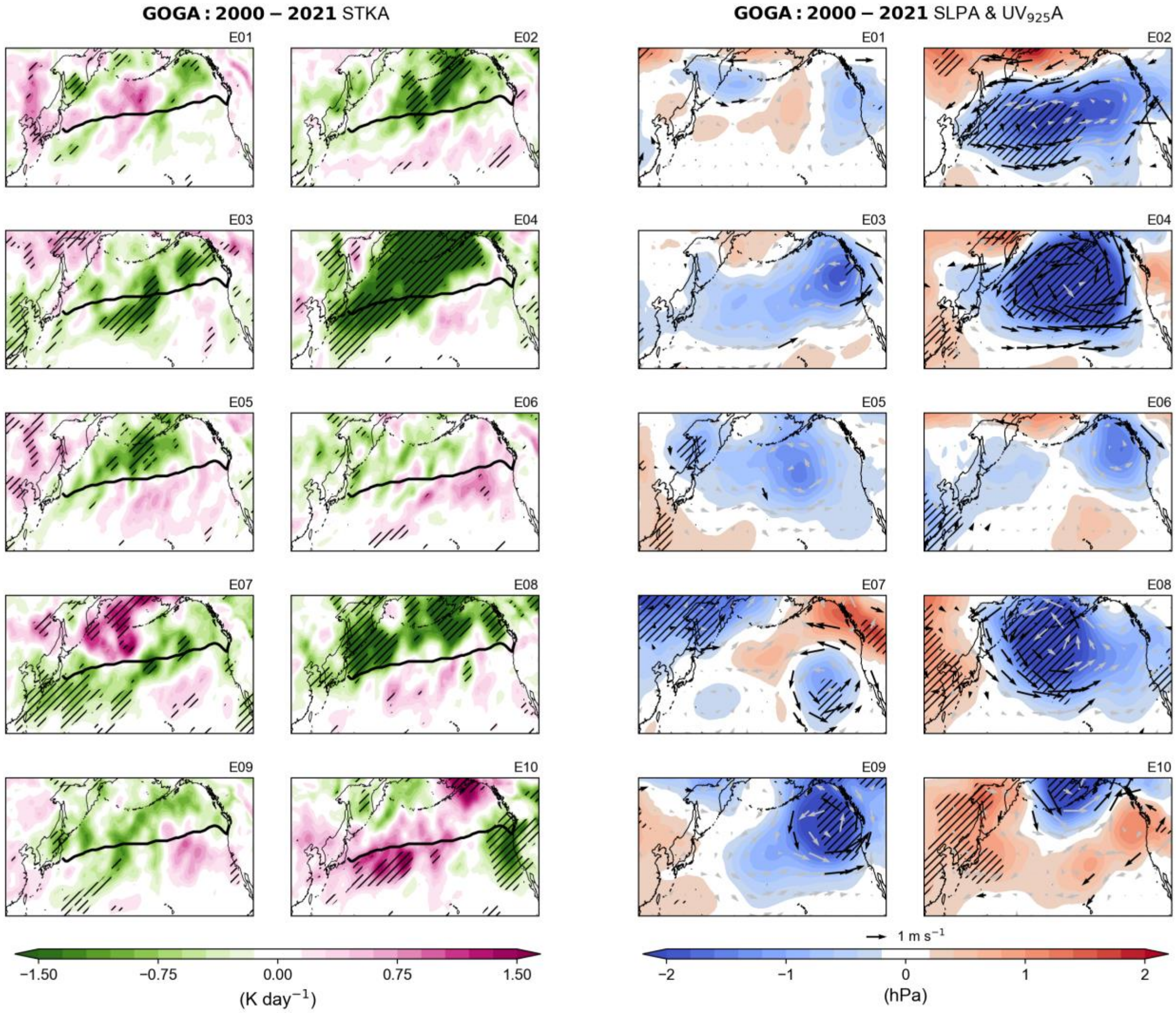

Fig. S17. Simulated KE atmospheric circulation patterns from individual ensemble members in the AGCM GOGA ensemble during 2000-2021. Same as fig. S16, but for AGCM GOGA ensemble during 2000-2021.

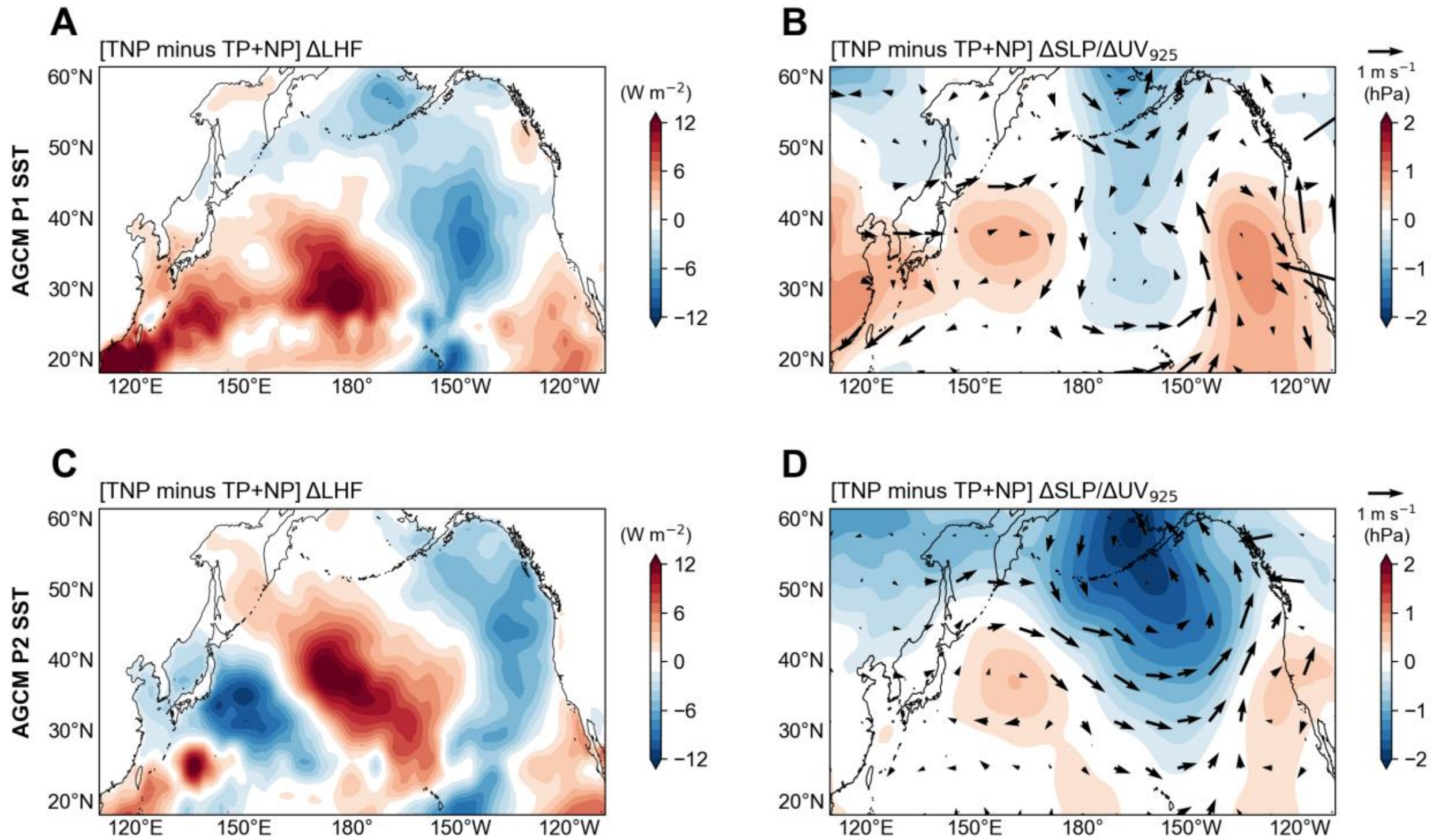

Fig. S18. Non-linear response in the LHF and atmospheric circulation patterns from AGCMs forced with SST patterns derived from P1 (1977-1999) and P2 (2000-2022). (**A,B**) Difference in the (**A**) LHF response (shading) and (**B**) SLP (shading) and 925-hPa wind (vectors) between the TNP SST-forced run and the linear sum of TP and NP SST-forced runs. (**C,D**), Same as (**A,B**), but for AGCMs forced with SST patterns derived from P2. In the figure titles, the Δ symbol denotes anomalies relative to the control simulations.

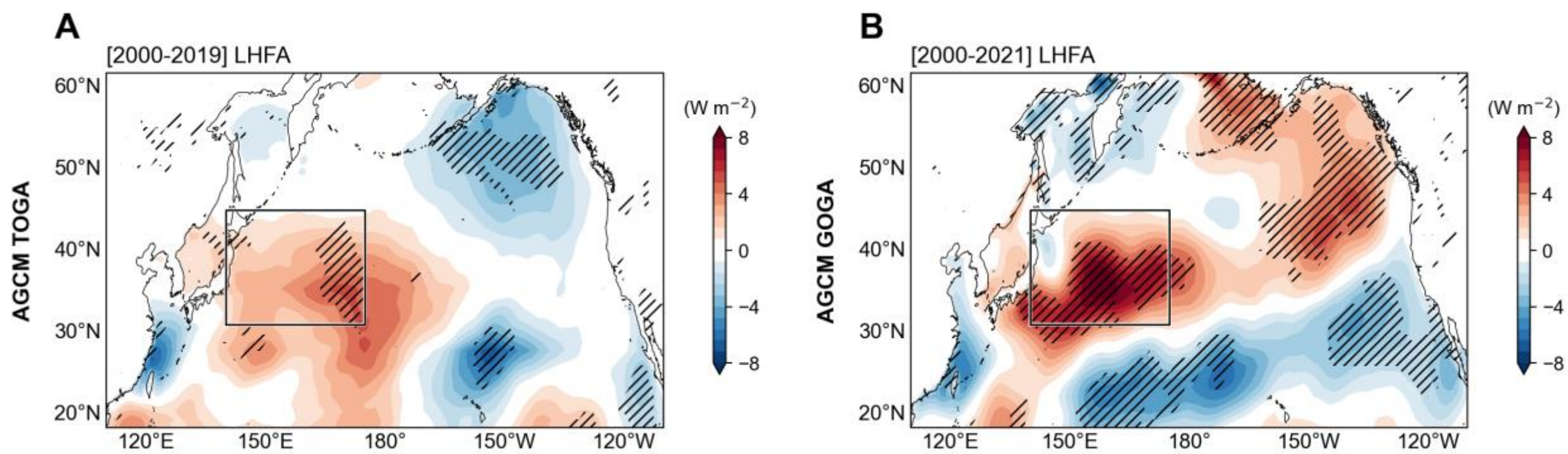

Fig. S19. Simulated LHF response from AGCM TOGA and GOGA ensemble. (**A,B**) Regression map of DJ LHF onto the SON KE index from (**A**) TOGA during 2000-2019 and (**B**) GOGA during 2000-2021, respectively. The black box denotes the KOE domain. Hatched areas indicate regions where at least 8 out of 10 ensemble members show the same sign in the regression coefficients. In the figure titles, the suffix "A" stands for anomaly.

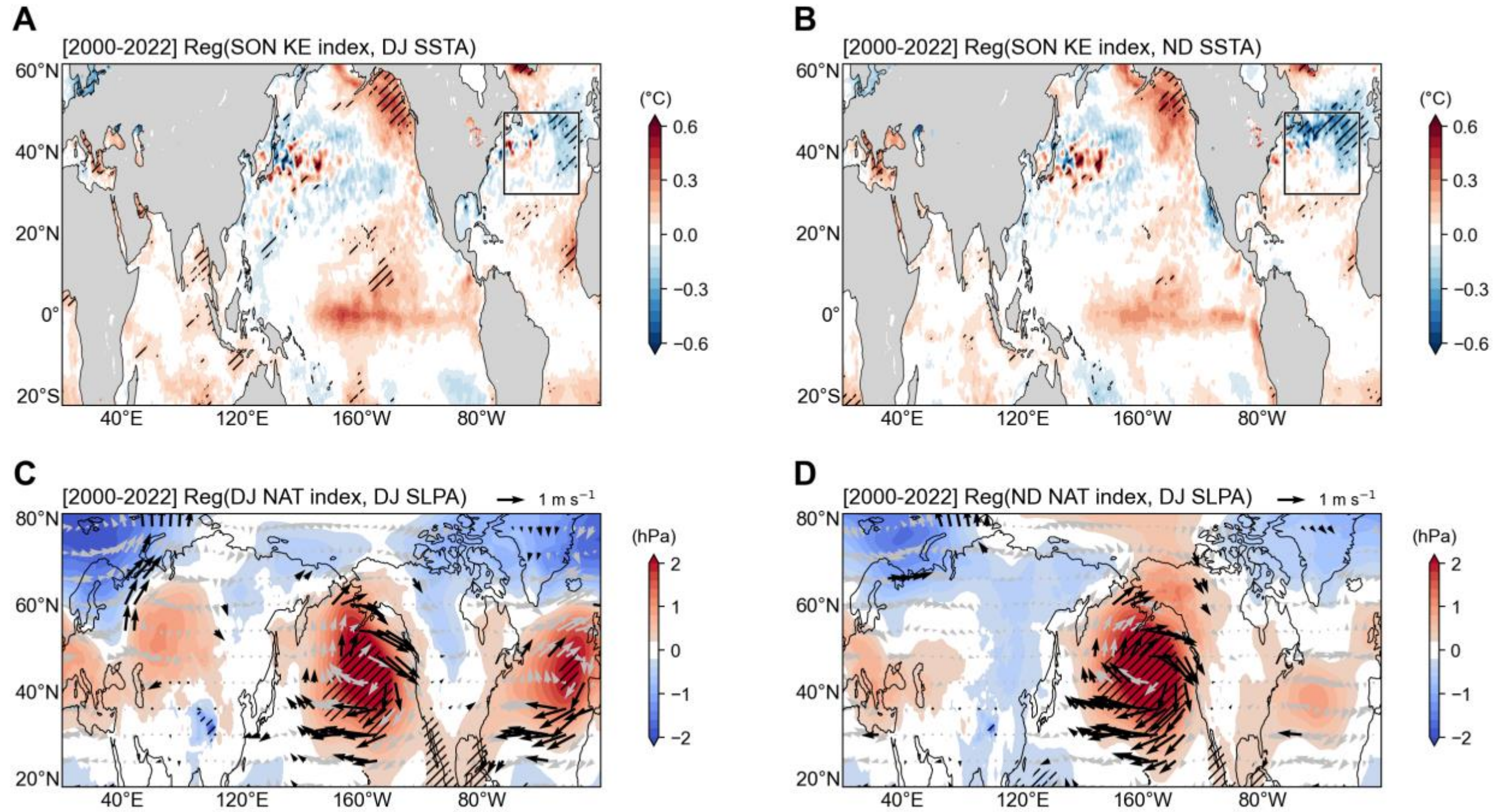

Fig. S20. Remote SST forcing from outside the tropical and North Pacific during 2000-2022. (**A,B**) Regression map of SST anomalies during (**A**) DJ and (**B**) ND onto the SON KE index during 2000-2022. (**C,D**) Regression map of DJ SLP anomalies onto the (**C**) DJ and (**D**) ND North Atlantic SST (NAT) index during 2000-2022. The black box in (**A,B**) denotes the North Atlantic domain (30°-50°N, 65°-15°W) used to define the NAT index. Hatched areas indicate regions with regression coefficients statistically significant at the 90% confidence level. In the figure titles, the suffix "A" stands for anomaly.

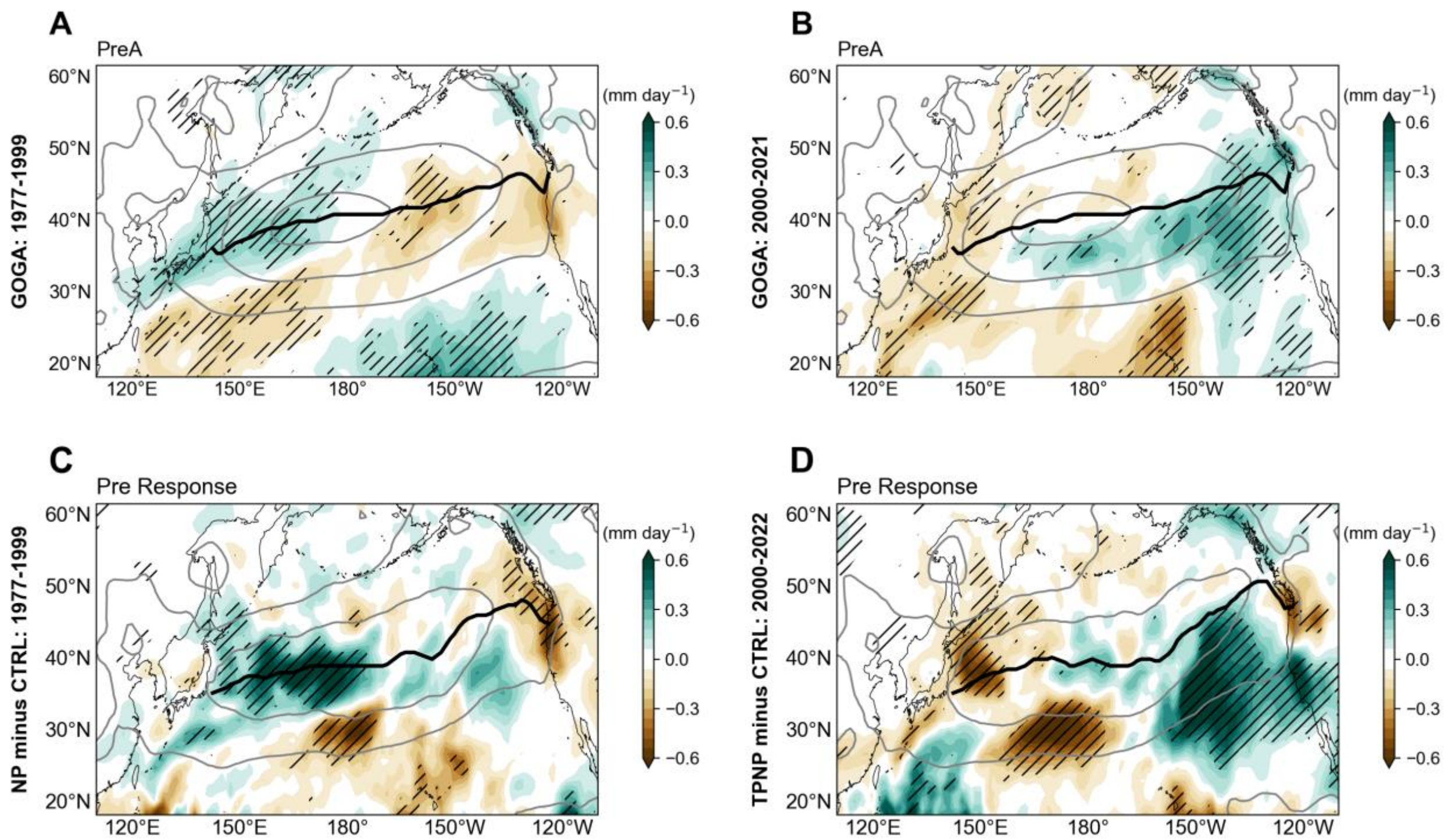

Fig. S21. Simulated precipitation response from AGCM GOGA, North Pacific, and combined tropical-North Pacific SST runs. (**A,B**) Regression map of DJ precipitation anomalies onto the SON KE index during (**A**) 1977-1999 and (**B**) 2000-2021 from the CESM2 GOGA simulation. (**C,D**) DJ precipitation response from (**C**) the North Pacific SST prescribed simulation in 1977-1999 and (**D**) the combined tropical-North Pacific SST prescribed simulation in 2000-2022 relative to the corresponding control simulation. The black line denotes the latitude of maximum storm track activity at each longitude over the North Pacific. Hatched areas in (**A,B**) indicate regions where at least 8 out of 10 ensemble members show the same sign in the regression coefficients, and in (**C,D**) indicate regions where the composite fields are statistically significant at the 90% confidence level. In the figure titles, the suffix “A” stands for anomaly.

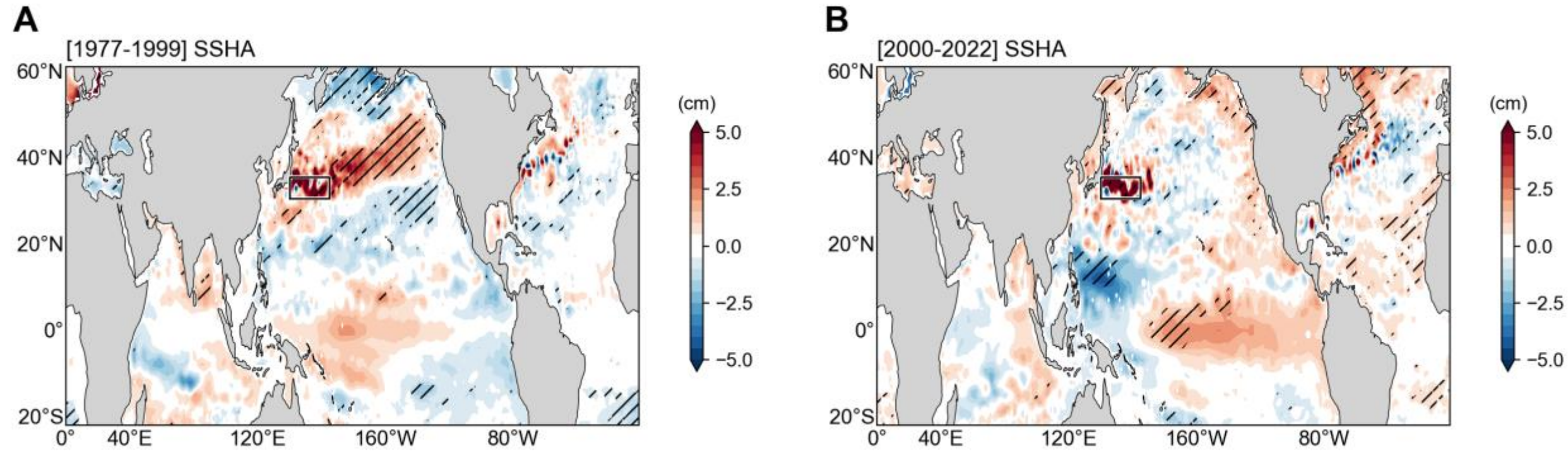

Fig. S22. SSH anomalies liked to KE variability. (**A,B**) Regression map of DJ SSH anomalies onto the SON KE index during (**A**) 1977-1999 and (**B**) 2000-2022. The black box denotes the KE domain (31°-36° N, 140°-165° E). Hatched areas indicate regions with regression coefficients statistically significant at the 90% confidence level. In the figure titles, the suffix “A” stands for anomaly.

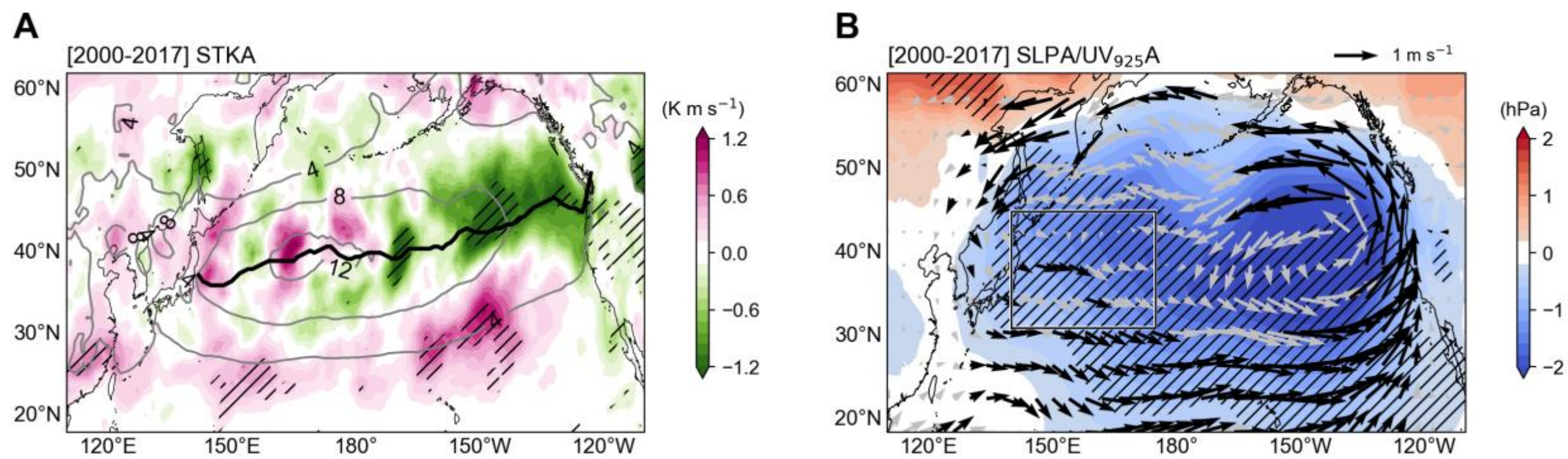

Fig. S23. Observed KE atmospheric circulation patterns from observational reanalysis during 2000-2017. (**A,B**) Regression map of DJ (**A**) storm track activity (shading) and (**B**) sea level pressure (SLP; shading) and 925-hPa wind (vector) onto the SON KE index during 2000-2017. The contours and black line in (**A**) denote the climatological DJ storm track activity and the latitude of maximum storm track activity at each longitude over the North Pacific, respectively. The black box in (**B**) denotes the KOE domain. Hatched areas (for shading) and black vectors (for wind) indicate regions where the regression coefficients are statistically significant at the 90% confidence level. In the figure titles, the suffix “A” stands for anomaly.

Table S1. Correlation between the KE SSH index and the KOE SST/LHF index during 1977-2022, 1977-1999, and 2000-2022.

Time period	KOE SST index	KOE LHF index
Total (1977-2022)	0.28*	0.40**
P1 (1977-1999)	0.61**	0.07
P2 (2000-2022)	0.07	0.66**

* and ** denote correlation coefficients significant at the 90% and 99% confidence levels, respectively.
